# RNA m^6^A Methylation Suppresses Insect Juvenile Hormone Degradation to Minimize Fitness Costs in Response to A Pathogenic Attack

**DOI:** 10.1002/advs.202307650

**Published:** 2023-12-12

**Authors:** Zhaojiang Guo, Yang Bai, Xinyi Zhang, Le Guo, Liuhong Zhu, Dan Sun, Kaiyue Sun, Xudan Xu, Xin Yang, Wen Xie, Shaoli Wang, Qingjun Wu, Neil Crickmore, Xuguo Zhou, Youjun Zhang

**Affiliations:** ^1^ State Key Laboratory of Vegetable Biobreeding Department of Plant Protection Institute of Vegetables and Flowers Chinese Academy of Agricultural Sciences Beijing 100081 China; ^2^ School of Life Sciences University of Sussex Brighton BN1 9QG UK; ^3^ Department of Entomology University of Kentucky Lexington Kentucky 40546‐0091 USA

**Keywords:** *Bacillus thuringiensis*, host‐pathogen interactions, juvenile hormone esterase, m^6^A modification, *Plutella xylostella*

## Abstract

Bioinsecticides and transgenic crops based on the bacterial pathogen *Bacillus thuringiensis* (Bt) can effectively control diverse agricultural insect pests, nevertheless, the evolution of resistance without obvious fitness costs has seriously eroded the sustainable use of these Bt products. Recently, it has been discovered that an increased titer of juvenile hormone (JH) favors an insect host (*Plutella xylostella*) to enhance fitness whilst resisting the Bt pathogen, however, the underlying regulatory mechanisms of the increased JH titer are obscure. Here, the involvement of *N* ^6^‐methyladenosine (m^6^A) RNA modification in modulating the availability of JH in this process is defined. Specifically, it is found that two m^6^A methyltransferase subunit genes, *PxMettl3* and *PxMettl14*, repress the expression of a key JH‐degrading enzyme JH esterase (JHE) to induce an increased JH titer, mitigating the fitness costs associated with a robust defense against the Bt pathogen. This study identifies an as‐yet uncharacterized m^6^A‐mediated epigenetic regulator of insect hormones for maintaining fitness during pathogen defense and unveils an emerging Bt resistance‐related m^6^A methylation atlas in insects, which further expands the functional landscape of m^6^A modification and showcases the pivotal role of epigenetic regulation in host‐pathogen interactions.

## Graphical abstract

This study found that increased expression of *PxMettl3* and *PxMettl14* in Bt‐resistant *P. xylostella* orchestrates the elevation of JH titer by reducing *PxJHE* expression via a key m^6^A modification site. This molecular mechanism increasing the JH titer in feeding larvae of Bt‐resistant *P. xylostella* compensates for the resistance‐related fitness costs.

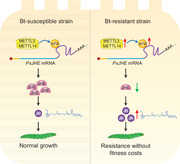

## 1 Introduction

As we know, one of the basic laws of nature is “survival of the fittest” within Darwinian evolutionary theory. During the long‐term co‐evolution between insect hosts and microbial pathogens, pathogens have evolved strong virulence to occupy their hosts for survival and reproduction, while hosts have developed immune defense mechanisms to repel these pathogens.^[^ ^1^ ^]^ Nonetheless, in the early stages of infection, the host defense mechanisms inevitably consume energy, which generally results in severe fitness costs, including slow growth, delayed development, low reproduction, and high mortality.^[^ ^2^ ^]^ However, some insect hosts can counteract the adverse effects to balance growth and defense, but how insect hosts gradually evolve and finally overcome pathogen infection with negligible fitness costs is largely unknown.^[^ ^3^ ^]^

*Bacillus thuringiensis* (Bt) is an environmentally friendly gram‐positive entomopathogenic bacterium that produces protein toxins (e.g., Cry and Vip toxins) that facilitate the infection of midgut cells of insect hosts, ultimately leading to their death.^[^ ^4^ ^]^ As a result, Bt sprays have become the most widely used microbial biopesticides and genetically modified crops expressing the toxins are extensively planted for pest control in agriculture.^[^ ^5^ ^]^ The long‐term coevolution between insects and Bt has resulted in defense responses against the pathogen as well as more extreme resistance phenotypes following prolonged exposure.^[^ ^6^ ^]^ The diamondback moth, *Plutella xylostella* (L.), is a worldwide cruciferous pest and is notable for being the first insect reported to develop resistance to Bt in the field.^[^ ^7^ ^]^ Thus, it has served as an excellent model for exploring the mechanism of insect resistance to Bt and its toxins. In our previous studies, we discovered that increased levels of both 20‐hydroxyecdysone (20E) and juvenile hormone (JH) activated a MAPK signaling pathway, which *trans*‐regulated the differential expression of multiple Bt toxin midgut receptors and nonreceptor paralogs by controlling the phosphorylation level of the transcription factor FTZ‐F1 to orchestrate high‐level resistance to the Bt Cry1Ac toxin in *P. xylostella*.^[^ ^8^ ^]^ We found that the increase in 20E titer was specifically linked to increased tolerance to the Bt Cry1Ac toxin through the downregulation of receptors, while the increased JH titer influenced the upregulation of non‐receptor paralogs which eliminated the fitness costs associated with receptor downregulation. However, the specific molecular mechanisms underlying the Bt toxin‐induced upregulation of hormone levels were unknown. In insects, the regulation of JH titer is very important for maintaining larval traits and preventing early metamorphosis, and research into the regulation mechanism of JH titer has mainly focused on this metamorphosis process.^[^ ^9^ ^]^ However, in *P. xylostella*, the Bt‐induced increase of JH titer occurs during the normal feeding stages and is thus clearly distinct from metamorphosis.

Epigenetic modifications have a significant impact on gene expression in a variety of critical biological processes.^[^ ^10^ ^]^ Recently, RNA methylation has emerged as an integral epigenetic modification that regulates the destiny of RNA molecules. To date, more than 170 RNA modifications have been identified, with *N* ^1^‐methyladenosine (m^1^A), 5‐methylcytosine (m^5^C), and *N* ^6^‐methyladenosine (m^6^A) in particular having been demonstrated to play significant roles in biological functions.^[^ ^11^ ^]^ Among them, m^6^A is the most prevalent modification present in cellular RNAs being found in virtually all types of RNA.^[^ ^12^ ^]^ m^6^A is a reversible RNA modification that can be dynamically controlled through m^6^A methyltransferases (writers), demethylases (erasers), and m^6^A‐binding proteins (readers). The “writers” and “erasers” can act together to regulate the abundance of m^6^A by installing or removing m^6^A modification, respectively.^[^ ^13^ ^]^ The “readers” can identify RNA molecules that have been modified with m^6^A to alter gene expression thereby orchestrating diverse physiological and pathological processes.^[^ ^14^ ^]^ Moreover, it has been proposed that m^6^A modification is involved in the human and plant immune responses against pathogen infections.^[^ ^15^ ^]^ However, whether m^6^A modification is involved in the molecular mechanisms of insect defense against pathogens is unclear.

In this study, we found that *PxMettl3* and *PxMettl14*‐mediated m^6^A modification negatively regulates the expression of PxJHE – a key metabolic enzyme modulating JH titer in *P. xylostella*. The elevated expression of *PxMettl3* and *PxMettl14* resulted in increased m^6^A level in *PxJHE* mRNA, reducing *PxJHE* gene expression and eventually leading to an upregulation of JH titer to combat Bt infection with growth balance. This finding defines the crucial role of m^6^A modification in hormonal regulation of growth‐defense tradeoffs during host‐pathogen interactions, which could be utilized for managing insect Bt resistance and developing novel pest control strategies.

## 2 Results

### 2.1 Midgut m^6^A Level is Elevated in Response to Bt Exposure

**Figure 1:**
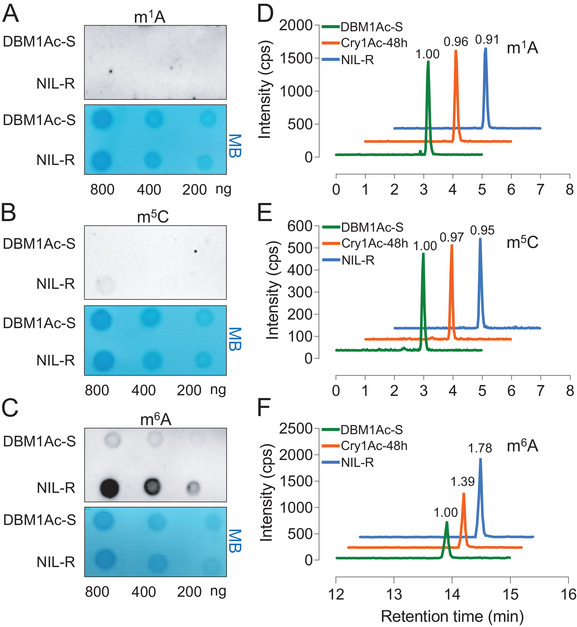
The detection of RNA modification levels in the midgut of Bt‐susceptible and ‐resistant *P. xylostella*. A–C) Dot blot assays were used to detect the modification levels of RNA (m^1^A, m^5^C, and m^6^A) extracted from the midguts of DBM1Ac‐S and NIL‐R strains. RNA samples were serially diluted and loaded equally with the amounts of 800, 400, and 200 ng. Methylene blue staining (lower blots) was used as a loading control to detect input RNA, while the intensity of the immunoblotting spot (upper blot) represented the level of each RNA modification. D–F) HPLC‐MS/MS assays were used to detect the modification levels of RNA (m^1^A, m^5^C, and m^6^A) extracted from the midgut of DBM1Ac‐S, DBM1Ac‐S Cry1Ac‐treated, and NIL‐R strains, the numbers above each peak indicates the relative m^6^A level calculated by the ratio of peak areas.

To explore whether m^1^A, m^5^C, and m^6^A, which have been known to participate in a wide range of vital physiological functions,^[^ ^16^ ^]^ might be associated with resistance to Bt Cry1Ac toxin in *P. xylostella*, we initially used dot blot assays to detect modification levels of m^1^A, m^5^C, and m^6^A in the midgut of Bt‐susceptible (DBM1Ac‐S) and near‐isogenic resistant (NIL‐R) strains of *P. xylostella*. Using methylation‐specific antibodies, no obvious signal could be detected for m^1^A or m^5^C, however, for m^6^A, a signal could be detected and was clearly higher in the resistant strain (**Figure** **1**A–C). To follow up on these preliminary dot blot assays, we used high‐performance liquid chromatography‐tandem mass spectrometry (HPLC‐MS/MS) to quantify each of these modifications in the midgut of susceptible, resistant, and Bt Cry1Ac‐treated susceptible strains. HPLC‐MS/MS assays confirmed that the m^6^A modification level was significantly increased in the midgut of both resistant and Bt Cry1Ac‐treated strains (Figure 1D–F). Thus, we speculated that m^6^A modification might be associated with the response of *P. xylostella* to Bt Cry1Ac.

### 2.2 *PxMettl3* and *PxMettl14* Genes are Upregulated in the Midgut

**Figure 2:**
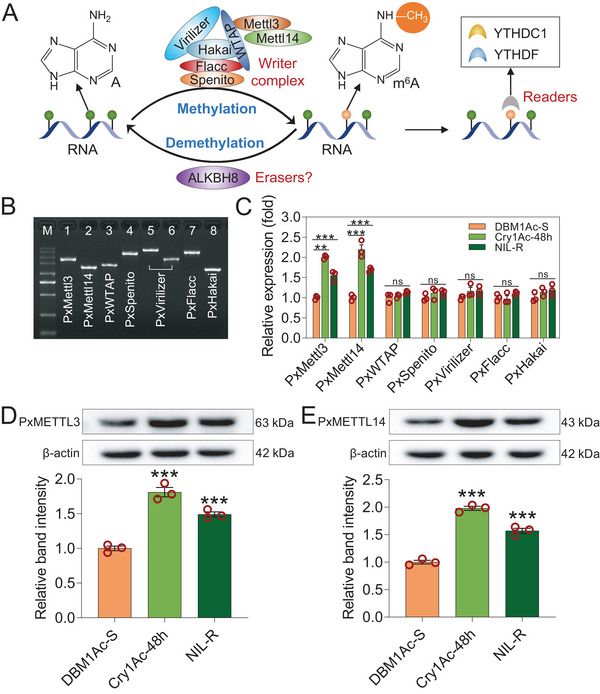
Cloning and characterization of *P. xylostella* m^6^A methyltransferase subunit genes. A) Schematic diagram of the molecular basis of RNA m^6^A modification in model insects. The m^6^A methyltransferase complex and a potential demethylase ALKBH8 recently identified in insects regulate m^6^A modification in RNA. The m^6^A reader proteins can bind to the modified RNA with m^6^A and mediate specific functions. B) Agarose gel electrophoresis of PCR amplification products of m^6^A methyltransferase subunit genes. M, marker (from top to bottom: 5000, 3000, 2000, 1500, 1000, 750, 500, 250, 100 bp); lanes 1–8, PCR products of the individual subunit genes (lanes 5 and 6, overlap‐extension PCR was used to clone the *PxVirlizer* gene). C) Relative expression levels of the m^6^A methyltransferase subunit genes in the midgut of Bt susceptible, Bt Cry1Ac‐treated, and Bt‐resistant *P. xylostella* strains. The relative expression levels of the genes have been normalized to the expression level of the internal control *RPL32* gene and the expression levels in the DBM1Ac‐S strain set to 1.0. D and E) The relative protein levels of PxMETTL3 (D) and PxMETTL14 (E) in the midgut of Bt susceptible, Bt Cry1Ac‐treated, and Bt‐resistant *P. xylostella* strains. The levels were detected by western blot, quantitated by densitometry, and normalized to β‐actin. Data were presented as mean values ± SEM (C–E), *n* = 3 biologically independent samples, *p < 0.05, **p < 0.01, ***p < 0.001, ns, not significant, one‐way ANOVA with Tukey's test was used for comparison.

As mentioned earlier, m^6^A modification is a reversible process regulated by methyltransferases and demethylases. In insects, ALKBH8 has been recently identified as a potential demethylase involved in this process (**Figure** **2**A).^[^ ^17^ ^]^ Since the *ALKBH8* gene encoding a demethylase homolog has not yet been confirmed in insects, we first cloned the m^6^A methyltransferase subunit genes *PxMettl3*, *PxMettl14*, *PxWTAP*, *PxSpenito*, *PxVirlizer*, *PxFlacc* and *PxHakai* (Figure 2B; Figure S1, Supporting Information) and evaluated their expression levels in the midgut of susceptible, resistant and Bt Cry1Ac‐treated strains. In line with the dot blot and HPLC‐MS/MS data, we found that the transcriptional levels of *PxMettl3* and *PxMettl14* were high in the resistant and Cry1Ac‐treated strains compared to the susceptible strain (Figure 2C). The protein levels of *PxMettl3* and *PxMettl14* were also significantly increased in these two strains (Figure 2D,E). Taken together, these data suggested that *PxMettl3* and *PxMettl14* were significantly overexpressed in the midgut of resistant and Bt Cry1Ac‐treated strains and that the increase of *PxMettl3* and *PxMettl14* might be associated with the response of *P. xylostella* to Bt Cry1Ac.

### 2.3 *PxMettl3* and *PxMettl14* Genes Regulate JH Titer

**Figure 3:**
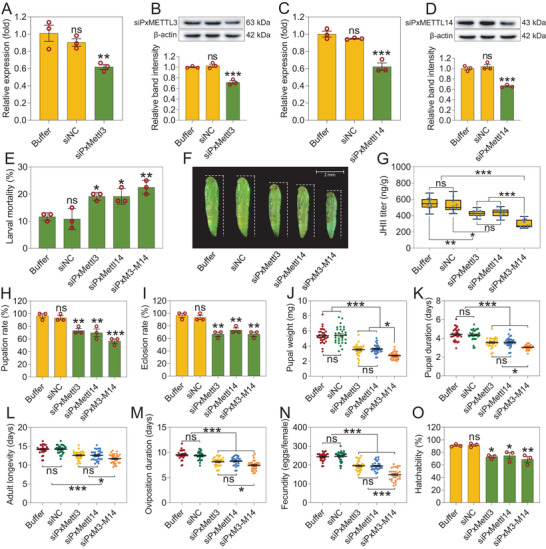
*PxMettl3* and *PxMettl14* regulate JH titer affecting the growth and development of *P. xylostella*. A‐‐D) siRNA‐mediated silencing of *PxMettl3* and *PxMettl14* were detected at both transcriptional (A,C) and protein (B,D) levels in the resistant NIL‐R strain. The relative expression levels of *PxMettl3* and *PxMettl14* have been normalized to that of the internal control *RPL32* gene and the expression level of buffer‐treated strain was set to 1.0. The protein levels were quantified by densitometry and normalized to β‐actin. E–O) Effect of silencing *PxMettl3*, *PxMettl14*, or both genes in NIL‐R resistant strain on larval susceptibility to an LC_10_ concentration of Cry1Ac protoxin (E) (1000 mg L^−1^), pupal morphology (F), JH titer (G), and fitness costs (H‐‐O) parameters. Data are presented as mean values ± SEM (A–E and G–O), *n* = 3 biologically independent samples (A‐‐E, H,I and O), *n* = 10 biologically independent samples (G), *n* = 30 biologically independent samples (J‐‐N), *p < 0.05, **p < 0.01, ***p < 0.001, ns, not significant, one‐way ANOVA with Tukey's test was used for comparison.

To further probe the possible role of m^6^A modification in the response of *P. xylostella* to Bt, we performed RNA interference (RNAi) mediated knockdown of *PxMettl3* and *PxMettl14*, or both (*PxM3‐M14*) in the resistant strain by microinjecting sublethal doses of gene‐specific small interfering RNAs (siRNAs). Both the transcript and encoded protein levels of both genes were significantly reduced at 48 h post‐injection (**Figure** **3**A–D). To determine if this knockdown affected the susceptibility *P. xylostella* to Bt Cry1Ac toxin, bioassays were performed at 72 h post‐using an LC_10_ concentration of 1000 mg L^−1^. A small, but significant, increase in mortality was observed in the siRNA‐treated larvae (Figure 3E). Although Cry1Ac‐induced mortality was only slightly increased following siRNA injection, the size of pupae was clearly smaller compared to controls (Figure 3F), suggesting that the silencing of *PxMettl3*, *PxMettl14*, or both (*PxM3‐M14*) impaired the growth and development of resistant *P. xylostella* larvae. As well as pupal size, we measured a number of other life history traits (pupation rate, eclosion rate, pupal weight, pupal duration, oviposition duration, fecundity, hatchability, and adult longevity) and found that each of these indicators of fitness was reduced under the three knockdown conditions (Figure 3H–O). Since we had previously associated JH titer with fitness, we used ultra‐performance liquid chromatography‐tandem mass spectrometry (UPLC‐MS/MS) to measure the JH titer in the resistant strain after knockdown of *PxMettl3*, *PxMettl14*, or both (*PxM3‐M14*). We found that the JH titer was significantly reduced in the resistant strain under all three knockdown conditions (Figure 3G). In conclusion, these findings support the idea that the higher JH titer in the resistant strain might be associated with the upregulation of *PxMettl3* and *PxMettl14* in the insect midgut, and therefore that the upregulation of these two genes ensures that Bt‐challenged *P. xylostella* maintains physiological fitness.

### 2.4 MeRIP‐seq Uncovers the Extent of m^6^A RNA Modification

**Figure 4:**
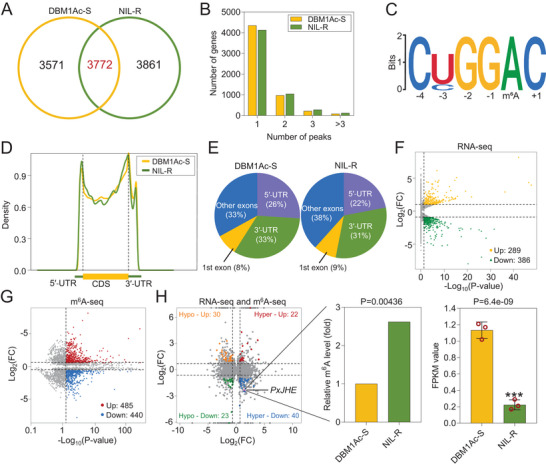
MeRIP‐seq and RNA‐seq analyses identify *PxJHE* as a gene potentially involved in regulating JH titer. A) The number of common and strain‐specific m^6^A peaks in the midgut of susceptible DBM1Ac‐S and resistant NIL‐R strains. B) The number of peaks of m^6^A‐modified genes identified in MeRIP‐seq analysis. C) Representative sequence motif identified from the 1000 most significant m^6^A peaks in the DBM1Ac‐S strain. D) Pie chart showing m^6^A peak distribution in the indicated gene regions from the midgut of DBM1Ac‐S and NIL‐R strains. E) Metagene profiles of m^6^A distribution across the transcriptome of the midgut of DBM1Ac‐S and NIL‐R strains. F) Volcano plot displaying differentially expressed genes from RNA‐seq analysis between DBM1Ac‐S and NIL‐R strains (Fold change > 2 and *p* < 0.05). G) MeRIP‐seq volcano plot showing genes with upregulated and downregulated m^6^A levels between DBM1Ac‐S and NIL‐R strains (Fold change > 2 and *p* < 0.05). H) Quadrant diagram representing the combined analysis of MeRIP‐seq and RNA‐seq. The insert boxes show individual modification and expression data for the identified *PxJHE* gene.

To explore the relationship between *PxMettl3* and *PxMettl14* and the fitness costs described above, we first obtained a transcriptome‐wide m^6^A map with methylated RNA immunoprecipitation sequencing (MeRIP‐seq) and RNA‐sequencing (RNA‐seq) in the midgut of Bt‐susceptible and ‐resistant *P. xylostella* strains. We identified 7343 m^6^A peaks representing the transcripts of 5631 genes (*p* < 0.05, fold enrichment > 5) in the susceptible strain, and 7633 m^6^A peaks representing transcripts of 5584 genes (*p* < 0.05, fold enrichment > 5) in the resistant strain. Among them, 3772 m^6^A peaks were common between the susceptible and resistant strains, and 3861 peaks were specific to the resistant strain (**Figure** **4**A). In addition, by analyzing the relationship between gene number and m^6^A peaks, we found that 77.3% of genes in the susceptible strain and 74.0% of genes in the resistant strain only possess one m^6^A peak, while a small fraction of genes possess two or more m^6^A peaks in both strains (Figure 4B). In order to ascertain if the m^6^A peaks that we identified contained the m^6^A consensus sequence of RRACH, we analyzed the 1000 most significant peaks in each of the six samples and confirmed that the consensus sequence was indeed conserved in *P. xylostella* (Figure 4C; Figure S2, Supporting Information). Subsequently, we analyzed the distribution pattern of m^6^A across the whole transcriptome of both strains and found that the majority of reads from m^6^A‐IP appeared in untranslated regions (UTRs) near the start and stop codons (Figure 4D). This phenomenon is similar to other organisms including mammals and yeast, suggesting that the distribution pattern of m^6^A is conserved. To further ascertain the preferential locations of m^6^A, the metagene profiles of m^6^A peaks were investigated and gave similar results (Figure 4E). From the RNA‐seq and MeRIP‐seq data analyses, a total of 675 differentially expressed genes and 925 differentially methylated genes were picked for further study (Figure 4F,G).

### 2.5 MeRIP‐seq and RNA‐seq Identify *PxJHE* as a Target Gene

To better understand how *PxMettl3* and *PxMettl14* could affect JH titer, we performed a combined analysis of MeRIP‐seq and RNA‐seq data (Figure 4H) and concentrated on transcripts that showed higher m^6^A levels and were differentially expressed in the resistant strain. One such transcript immediately presented itself as a candidate for the control of JH titer – a gene annotated as juvenile hormone esterase (JHE) [NCBI Gene ID: LOC105387899 (Figure S3, Supporting Information)]. We found a circa 5‐fold decrease in its transcript level and a circa 3‐fold increase in its level of m^6^A modification in the midgut of the resistant strain compared to the susceptible one (Figure 4H). JHE is well known to be involved in the JH degradation pathway, metabolizing JH into juvenile hormone acid (JHA) – a critical physiological process for insect growth and development.^[^ ^18^ ^]^ This encouraging result prompted us to test whether the rise in JH titer in the resistant strain was due to a decrease in *PxJHE* expression in the midgut.

### 2.6 JHE Affects Larval Fitness via Reducing JH Titer

**Figure 5:**
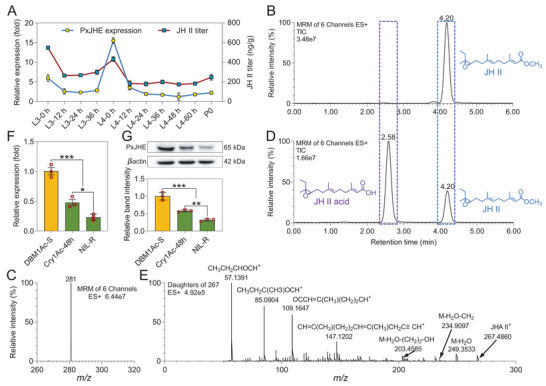
PxJHE can metabolize JH and its expression is reduced by Bt Cry1Ac‐treatement. A) *PxJHE* expression level and JH II titer from the third‐ to fourth‐instar larvae of the susceptible DBM1Ac‐S strain. Samples were collected every 12 h. B–E) Metabolic analyses of heterologously expressed PxJHE protein by UPLC‐MS/MS. Control group without heterologously expressed protein (B). Treatment group with heterologously expressed protein (D). Representative ion chromatographs of JH II (C) and JH II acid (E) are shown. F,G) The transcriptional (F) and protein (G) levels of *PxJHE* in the midgut of susceptible, Bt Cry1Ac‐treated, and resistant *P. xylostella* strains. The expression level of the *PxJHE* has been normalized to the expression level of the internal control *RPL32* gene and the expression level of the DBM1Ac‐S strain was set to 1.0. The protein level was detected by western blot, quantitated by densitometry, and normalized with β‐actin. Data are presented as mean values ± SEM (A,F, and G). *n* = 3 biologically independent samples, *p < 0.05, **p < 0.01, ***p < 0.001, ns, not significant, one‐way ANOVA with Tukey's test was used for comparison.

We first wanted to confirm that the gene annotated as *PxJHE* was a functional and physiologically relevant *JHE*. To do this, we first performed a detailed expression analysis of this gene to explore whether it exhibited the characteristic developmentally related changes in expression. We confirmed such a change during the larval molting stage where a spike in expression correlated with a drop in JH titer in a time‐lagged manner (**Figure** **5**A). To further confirm that the gene encodes a functional JHE enzyme, we decided to heterologously express the protein and check that it could metabolize JH. Its full‐length cDNA sequence was cloned into the pFastBac1 expression vector and used to transfect Sf9 cells (Figure S4, Supporting Information). We found that compared with the control group lacking heterologously expressed protein, the expressed PxJHE caused the signal peak of JH to decrease and a new signal for JHA to emerge (Figure 5B–E). qPCR and western blot data then showed that both transcript and protein levels of *PxJHE* were decreased in both the resistant strain and in the Cry1Ac‐treated susceptible one (Figure 5F,G).

**Figure 6:**
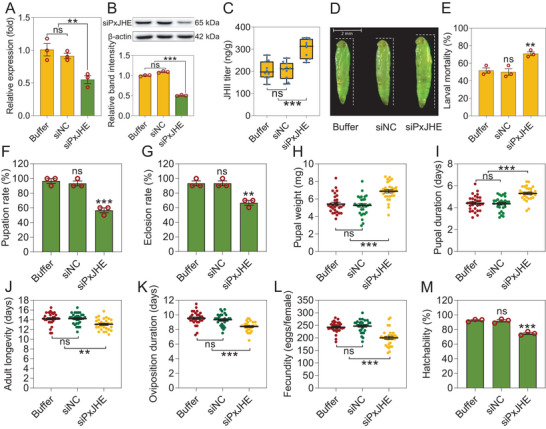
Silencing of the *PxJHE* gene affects the growth and development of *P. xylostella*. A,B) siRNA‐mediated silencing of *PxJHE* was detected at the transcriptional (A) and protein (B) levels in the susceptible DBM1Ac‐S strain. The expression level of *PxJHE* has been normalized to the expression level of the internal control *RPL32* gene and the expression level of buffer‐treated DBM1Ac‐S larvae was set to 1.0. The protein level of *PxJHE* was quantitated by densitometry and normalized with β‐actin. C–M) Effect of silencing *PxJHE* gene expression in the susceptible DBM1Ac‐S strain on JH II titer (C), pupal morphology (D), larval susceptibility to LC_50_ concentration of Cry1Ac protoxin (1 mg L^−1^) (E), and fitness cost traits (F‐‐M). Data are presented as mean values ± SEM. *n* = 3 biologically independent samples (A,B,E–G, and M), *n* = 10 biologically independent samples (C), *n* = 30 biologically independent samples (H‐‐L), *p < 0.05, **p < 0.01, ***p < 0.001, ns, not significant, one‐way ANOVA with Tukey's test was used for comparison.

To confirm whether *PxJHE* could affect growth and development via controlling JH titer in *P. xylostella*, we silenced its expression in susceptible *P. xylostella* by siRNA (**Figure** **6**A,B). As expected, we found JH titer was significantly increased in silenced *P. xylostella* (Figure 6C). This knockdown of *PxJHE* also led to larvae developing into slightly larger pupae (Figure 6D). We also found some other significant changes in biological parameters of life‐history traits, specifically decreases in pupation rate, eclosion rate, adult longevity, oviposition duration, fecundity, and hatchability alongside increases in pupal weight and pupal duration (Figure 6F–M). A small increase in larval mortality was observed when treated with Bt Cry1Ac toxin (Figure 6E). Together, these results indicate that the decrease in *PxJHE* expression in the midgut of the susceptible strain elevates JH titer, affecting larval growth and development in *P. xylostella*. However, it was still not clear exactly how *PxMettl3* and *PxMettl14* regulate *PxJHE* expression.

### 2.7 *PxMettl3* and *PxMettl14* Regulate *PxJHE* via m^6^A Modification

**Figure 7:**
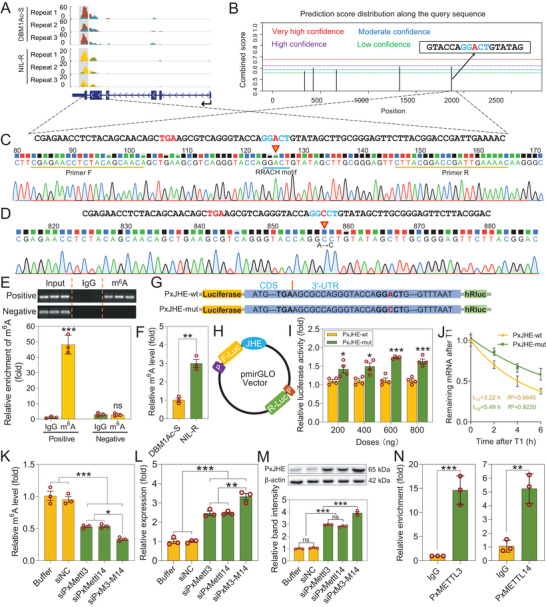
*PxMettl3* and *PxMettl14* regulate *PxJHE* expression by m^6^A modification. A) Visualization of m^6^A peaks on *PxJHE* mRNA using IGV software. B) Bioinformatic prediction of m^6^A site of *PxJHE*. C,D) Representative direct sequencing chromatograms of the PCR products containing m^6^A site of the wild type (C) and mutant (D) genes. E) m^6^A site of *PxJHE* was verified by m^6^A‐IP‐qPCR assays. F) m^6^A‐IP‐qPCR analysis of m^6^A level in the midgut of susceptible DBM1Ac‐S and resistant NIL‐R strains. G,H) Schematic diagrams of the pmirGLO vector containing PCR fragment of the *PxJHE* gene. I) Luciferase activity following transfection with varying amounts of the reporter plasmid. J) RNA lifetimes for *PxJHE*‐wt and *PxJHE*‐mut in S2 cells were determined by monitoring transcript abundance after transcription inhibition (TI). K) The m^6^A level was assessed in siPxMettl3, siPxMettl14, and siM3‐M14 strains by m^6^A‐IP‐qPCR. L,M) Effect of silencing *PxMettl3*, *PxMettl14*, or both genes on *PxJHE* mRNA expression (L) and protein (M) levels in the resistant NIL‐R strain. N) RIP‐qPCR showing enrichment of *PxJHE* mRNA by the *PxMettl3* and *PxMettl14* antibodies respectively. Data are presented as mean values ± SEM, *n* = 3 biologically independent samples (E,F, and J‐‐N), *n* = 4 biologically independent samples (I), *p < 0.05, **p < 0.01, ***p < 0.001, ns, not significant, one‐way ANOVA with Tukey's test was used for comparison.

Because the MeRIP‐sequencing data showed an m^6^A peak associated with *PxJHE* mRNA (**Figure** **7**A), we supposed that *PxMettl3* and *PxMettl14* might regulate *PxJHE* expression through m^6^A modification. Since the m^6^A mapping approaches used only offered a mediocre resolution (∼100‐200 nt), we could not identify the precise location of the m^6^A site. To identify the precise m^6^A site, we first attempted to use a bioinformatic prediction method. This revealed a GG(m^6^A)C motif in the 3′‐UTR near the stop codon on the *PxJHE* mRNA, which overlapped with the m^6^A peak from our MeRIP‐sequencing data (Figure 7B). We next verified the reliability of this prediction by using m^6^A‐IP‐qPCR assays. Two pairs of specific primers were designed for validation, one pair amplified this putative m^6^A‐containing region (Figure 7C), while the other pair amplified a region of exon 1 not shown to contain any m^6^A. The result of the m^6^A‐IP‐qPCR assays showed that the putative m^6^A site was robustly enriched by an m^6^A‐specific antibody, compared to the control IgG antibody, whereas no enrichment was seen with the exon 1 region (Figure 7E). We next explored whether there was a notable difference in the m^6^A level of *PxJHE* mRNA between the midgut of susceptible and resistant strains by employing m^6^A‐IP‐qPCR. In line with the MeRIP‐sequencing result, the m^6^A level of *PxJHE* mRNA in the midgut of the resistant strain was significantly increased about 3‐fold compared to the susceptible strain (Figure 7F). This compares to the 5‐fold decrease in *PxJHE* transcript level described earlier (Figure 4H).

The above results prompted us to explore whether m^6^A modification of *PxJHE* mRNA negatively regulates its expression. Recent studies have reported that m^6^A modification can accelerate mRNA decay, thereby reducing gene expression levels.^[^ ^19^ ^]^ To explore this, we mutated the m^6^A site by site‐directed mutagenesis changing adenosine (A) to cytosine (C) (Figure 7D). Then, we cloned *PxJHE*‐wt and *PxJHE*‐mut into the pmirGLO dual‐luciferase reporter vector to explore the function of this m^6^A site (Figure 7G). The dual‐luciferase report assays showed that luciferase activity was significantly increased when the m^6^A site was mutated (Figure 7I). In addition, we also cloned *PxJHE*‐wt and *PxJHE*‐mut into the pAc5.1/V5‐His B vector to perform an RNA decay assay to ascertain whether m^6^A regulates *PxJHE* mRNA stability. The results showed that the m^6^A mutation significantly increased the half‐life of *PxJHE* mRNA (Figure 7J). Taken together, these data indicated that *PxMettl3* and *PxMettl14* negatively regulate *PxJHE* expression through m^6^A modification. In order to substantiate this in vivo, we silenced *PxMettl3*, *PxMettl14*, and both (*PxM3‐M14*) expression by siRNA and then measured *PxJHE* expression levels. We observed that the m^6^A level of *PxJHE* mRNA was obviously decreased but that its expression was significantly increased in each knockdown, indicating that *PxMettl3* and *PxMettl14* negatively regulate *PxJHE* expression through m^6^A modification (Figure 7K*–*M). To investigate whether PxMETTL3 and PxMETTL14 directly regulate *PxJHE* expression, RNA immunoprecipitation following qPCR (RIP‐qPCR) was performed. The RIP‐qPCR assays showed that the *PxJHE* mRNA was significantly enriched by both PxMETTL3 and PxMETTL14 antibodies, suggesting that PxMETTL3 and PxMETTL14 can directly interact with *PxJHE* mRNA (Figure 7N).

### 2.8 Site‐Specific Mutation of the m^6^A Site in *PxJHE* Causes Serious Fitness Costs

**Figure 8:**
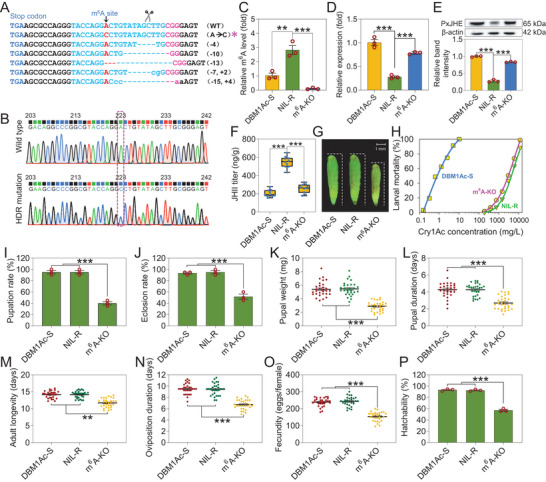
CRISPR/Cas9‐induced m^6^A site‐specific mutation of *PxJHE* induces fitness costs. A) Different site‐specific mutation types flanking the m^6^A site‐specific sgRNA target site in G_1_ larvae were identified by sequencing PCR products. B) Representative chromatograms of direct sequencing of PCR products derived from wild type (upper graph), HDR mutation (lower graph) in G_2_ individuals. The mutation from “A” to “C” in *PxJHE* sequence is highlighted by pink dotted lines. C) m^6^A‐IP‐qPCR analysis of m^6^A level in the midgut of DBM1Ac‐S, NIL‐R, and m^6^A‐KO strains. D,E) Effect of site‐specific mutation of m^6^A site on *PxJHE* mRNA expression (D) and protein (E) levels in the resistant NIL‐R strain. F‐P) Effect of site‐specific mutation of m^6^A site in NIL‐R resistant strain on JH titer (F), pupal morphology (G), larval susceptibility (H), and biological parameters of fitness costs (I‐‐P). Data are presented as mean values ± SEM. *n* = 3 biologically independent samples (C–E,I,J and P), *n* = 10 biologically independent samples (F), *n* = 30 biologically independent samples (I‐‐P), *p < 0.05, **p < 0.01, ***p < 0.001, ns, not significant, one‐way ANOVA with Tukey's test was used for comparison.

The CRISPR/Cas9 genome editing system is a powerful tool for in vivo exploration of gene functions in diverse insects.^[^ ^20^ ^]^ In order to further verify the function of the m^6^A site of *PxJHE* in vivo, we created a site‐specific mutation of the m^6^A site in *PxJHE* by CRISPR/Cas9‐mediated homology‐directed repair (HDR) pathway in *P*. *xylostella* (Figure S6A, Supporting Information). By this method, a homozygous precise m^6^A mutant strain m^6^A‐KO was established from the NIL‐R strain (**Figure** **8**A,B; S6B, Supporting Information). Subsequently, m^6^A‐IP‐qPCR was used to confirm that the m^6^A site in *PxJHE* had been successfully mutated (Figure 8C). Both the transcript (Figure 8D) and protein (Figure 8E) levels of *PxJHE* were increased in the m^6^A‐KO strain compared to the NIL‐R strain, whereas the JH titer was significantly reduced (Figure 8F). Furthermore, we found that the pupae size was obviously smaller (Figure 8G). Subsequent bioassay results showed that the mutation of the m^6^A site of *PxJHE* resulted in a slight increase in the susceptibility to Cry1Ac protein (Figure 8H). Additionally, we found that the reduced JH titer induced by m^6^A site‐specific mutation of *PxJHE* caused serious fitness costs compared to the DBM1Ac‐S and NIL‐R strains (Figure 8I*–*P).

## 3 Discussion

**Figure 9:**
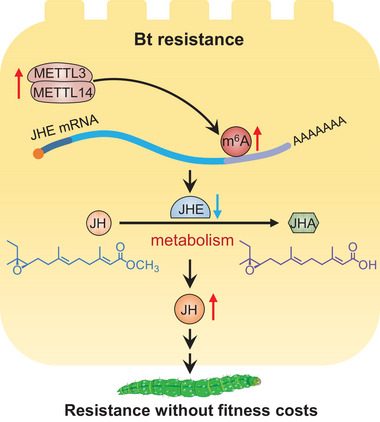
Summary model showing that m^6^A modification serves as a key regulator in *P. xylostella*’s defense against Bt pathogens. The expression levels of *PxMettl3* and *PxMettl14* are significantly increased in the midgut of Bt Cry1Ac‐treated or resistant *P. xylostella*. The elevation of both m^6^A methyltransferase subunits increases the m^6^A level of the target *PxJHE* gene, which encodes a key JH degrading enzyme within the JH metabolic pathway. The *PxMettl3* and *PxMettl14*‐mediated m^6^A modification negatively regulates the expression of the *PxJHE* gene, thus decreasing the expression of *PxJHE*, ultimately resulting in the elevation of JH titer. This drives a compensatory mechanism to counter fitness costs associated with the pathogen defense.

For host insects, the ability to defend against pathogens without causing serious physiological defects is crucial for their survival and reproduction and is also a sign of the success of host adaptive evolution. In this study, we revealed a molecular mechanism of adaptive evolution of the host insect *P. xylostella*, which prevents it from suffering severe fitness costs when defending against Bt pathogens. We found that *PxMettl3* and *PxMettl14*‐mediated m^6^A modification negatively regulates *PxJHE* gene expression, finally resulting in an elevation of JH titer to mitigate the costs of mounting the pathogen response (**Figure** **9**).

Upon exposure to Bt toxins, or in the resistant strain, the elevation of *P. xylostella* JH titer is attributed to the increase of *PxMettl3* and *PxMettl14* expression levels in the larval midgut. Both *PxMettl3* and *PxMettl14* play extremely important roles in regulating gene expression via m^6^A modification affecting various physiological functions.^[^ ^21^ ^]^ Research on transmethylase subunit genes in insects has mainly focused on the model insect *Drosophila melanogaster* and has indicated that they play essential roles in early embryogenesis, reproductive development, nervous system development, and sex determination.^[^ ^22^ ^]^ In our study, relatively high expression levels of transmethylase subunit genes were found in eggs, male and female adults, head, and testes in *P. xylostella* (Figure S5A,B, Supporting Information), suggesting that *PxMettl3* and *PxMettl14* may also play important roles in early embryogenesis, reproductive development, and nervous system development in *P. xylostella*. Little research has been done on other physiological functions of transmethylase subunit genes in insects. As mentioned earlier, m^6^A modification is extremely abundant in living organisms, and m^6^A modification participates in various important physiological functions. Consequently, abnormal regulation of m^6^A modification can cause serious physiological dysfunction. In our study, we noted that whole‐body *PxMettl3* and *PxMettl14* expression did not vary significantly between the resistant and susceptible strains (Figure S5C,D, Supporting Information), but that the expression levels of *PxMettl3* and *PxMettl14* in the midgut of the resistant strain were 1.5 and 2 times higher than the susceptible strain, respectively. The expression levels of *PxMettl3* and *PxMettl14* in the midgut tissue of *P. xylostella* were, however, relatively low, particularly when compared to the head (Figure S5A,B, Supporting Information), which would explain why the whole‐body differences are negligible. The slight upregulation of *PxMettl3* and *PxMettl14* expression in the midgut could represent a tissue‐specific adaptive response to pathogens, which doesn't significantly impact the role of JH in other physiological processes.

Hormones play critical roles in helping animals and plants adapt to adverse environmental conditions.^[^ ^8^, ^23^ ^]^ Recent studies have suggested that epigenetic modifications such as microRNAs, DNA methylation, histone modification, and chromatin remodeling play a vital role in the regulation of these hormones.^[^ ^24^ ^]^ It has been found that DAA, an inhibitor of m^6^A methylation, can affect the JH titer and thus regulate development and caste differentiation in honeybees, although the exact molecular mechanism is unclear.^[^ ^25^ ^]^ In this study, we show how m^6^A modification regulates the JH titer of an insect, enhancing the adaptability of *P. xylostella* by contributing to its defense mechanism against Bt pathogens. JH is a key hormone in the control of the life history of insects, both in directing reproductive maturation and maintaining the larval state during molts.^[^ ^9a^ ^]^ JHE is a key enzyme for regulating JH degradation, playing an important role in the fine regulation of JH titers.^[^ ^18^ ^]^

We previously demonstrated that both JH and 20E hormones have a role to play in the defense of *P. xylostella* against Bt.^[^ ^8b^ ^]^ An increase in JH titer was found to induce the expression of a number of proteins expressed by the midgut endothelial cells, some of which acted as receptors for Bt Cry toxins. In contrast, an increase in 20E resulted in a downregulation of the proteins, however, a coordinated increase of both hormones saw a downregulation of the receptor proteins alongside an upregulation of non‐receptor paralogs. This combination allows the insect to resist Bt toxins by removing their receptors, while the paralogs ensure physiological fitness. The role of 20E is predominantly to control the downregulation of the receptors, while JH controls the upregulation of the non‐receptor paralogs. In this study, the effects on the fitness of silencing JHE (Figure 6F–M) exactly mirrored our previously reported effect of adding exogenous methoprene (a JH analog),^[^ ^8b^ ^]^ which could be expected as reducing the levels of JHE should increase the JH titer. Since we have demonstrated that m^6^A modification of *PxJHE* resulted in its downregulation, one might expect that silencing of the methyltransferase subunits *PxMettl3* and *PxMettl14* would have the inverse effect on the measured life history traits. This was not the case though since their silencing resulted in a reduction in all traits (Figure 3H,O). The methyltransferase containing these subunits will target many other RNA molecules, some of which are also likely to have effects on insect fitness. In terms of susceptibility to Bt Cry1Ac, silencing of *PxJHE* resulted in a slight increase in susceptibility (Figure 6E), which is consistent with our previous work where an increase in 20E was the main determinant of hormone‐induced tolerance.^[^ ^8b^ ^]^ Silencing of the methyltransferase subunits also resulted in a slight increase in susceptibility (Figure 3E), which again probably reflects their pleiotropic effects.

A possible explanation for the downregulation of m^6^A‐modified *PxJHE* is that the methylation triggered a degradation pathway. Two rapid degradation pathways of m^6^A‐containing mRNAs have been revealed: YTHDF2 (an m^6^A reader protein)‐mediated deadenylation via recruiting the CCR4/NOT (deadenylase) complex and YTHDF2‐mediated endoribonucleolytic cleavage by binding to the HRSP12‐RNase (ribonuclease) P/MRP (endoribonuclease) complex.^[^ ^19^ ^]^ Because the deadenylation‐dependent decay pathway depends on the shortening of the poly (A) tail within the 3′‐UTR,^[^ ^26^ ^]^ and the m^6^A site of *PxJHE* mRNA is located 18 bp downstream of the translational stop codon in the 3′‐UTR, we speculate that m^6^A‐mediated negative regulation of *PxJHE* may be due to YTHDF2‐mediated deadenylation. However, in insects, only one YTHDF2 homologous protein has been identified,^[^ ^27^ ^]^ and whether the YTHDF in insects can mediate the deadenylation of m^6^A‐containing mRNAs is unclear. Thus, the specific mechanism underlying how the m^6^A site negatively regulated *PxJHE* expression needs further investigation.

In this article, we dissected the role of m^6^A modification in host adaptive evolution. All living organisms live in a complex environment and are constantly affected by multiple environmental factors. Adaptability is the most basic ability that organisms have to survive in a varied environment. If a species lacks the necessary ability to adapt to change, it may be eliminated by natural or artificial selection, leading to species extinction,^[^ ^28^ ^]^ Studies have shown that genome dynamics is the major factor affecting the adaptive evolution of species, including the activation of transposable elements, induction of mutations, and an increase in chromosome number.^[^ ^29^ ^]^ Apart from those, increasing evidence suggests that epigenetic inheritance plays an important role in adaptive evolution, including DNA methylation, histone acetylation, and histone methylation.^[^ ^30^ ^]^ To date, research on epigenetics in adaptive evolution has mainly concentrated on DNA and histone modifications. What role m^6^A, the most abundant internal RNA modification, has in the adaptive evolution of living organisms is less clear. However, a large number of studies have shown that m^6^A modification can serve as a regulatory factor for living organisms to adapt to adverse conditions. For instance, in animals, the immune system is a defense mechanism gradually established by contact with the changing environment in the process of long‐term evolution. It is a markedly important line of animal defense against pathogen infection, and also one of the landmark events of animal adaptive evolution.^[^ ^31^ ^]^ A growing body of evidence suggests that m^6^A modification can act as a novel regulator in the human immune response, including immune system development, immune recognition, the induction of innate and adaptive immune responses, and immune cell fate determination.^[^ ^15a^ ^]^ Unlike animals, plants have no ability to move, so they must endure a large number of biotic and abiotic stresses in their environment such as extreme temperatures and pathogenic infection. These stressors inevitably limit the distribution of plants, reduce their rate of growth and development, and decrease crop productivity.^[^ ^32^ ^]^ Thus, it is very important for plants to adapt to environmental stresses. Substantial evidence has identified m^6^A modification as a key regulatory factor involved in the defense response against pathogen infection and adaption to heavy metal stress, water and drought stresses, salt stress, and extreme temperatures.^[^ ^15b^ ^]^ In insects, we have recently found that m^6^A modification can regulate a cytochrome P450 gene *CYP4C64* thereby conferring resistance to the neonicotinoid insecticide thiamethoxam in *Bemisia tabaci*.^[^ ^33^ ^]^ For pathogenic microorganisms, evading the host's immune defenses is essential for survival. A study showed that m^6^A modification of genomic RNAs of human metapneumovirus is important for evading immune recognition.^[^ ^34^ ^]^ Since December 2019, Coronavirus Disease 2019 (COVID‐19), caused by a novel coronavirus named Severe Acute Respiratory Syndrome coronavirus 2 (SARS‐CoV‐2), has spread throughout the world in short order and become a pandemic. A study showed that epidemic strains with mutations at identified m^6^A motifs have been found worldwide. Mutation of m^6^A motifs may be a strategy for virus infection and survival.^[^ ^35^ ^]^

Through the above studies and our results, we can surmise that m^6^A modification can regulate the adaptive ability of organisms to adverse environments in different ways or pathways in different species. The reason for this may be that m^6^A can serve as a key regulator that enables the cell to respond to different environmental stimuli, which can endow living organisms with an ability to constantly adapt in a challenging environment. In conclusion, we propose that m^6^A modification can be used as an evolutionary factor to regulate host adaptive evolution by fine‐tuning insect hormone levels, which provides a novel molecular target for insect resistance management to Bt products.

## 4 Experimental Section

### Insect Strains and Cell Lines

In this study, two *P. xylostella* strains were used, one Bt Cry1Ac susceptible strain (DBM1Ac‐S) and one Bt Cry1Ac near‐isogenic resistant strain (NIL‐R) constructed by crossing the susceptible DBM1Ac‐S and resistant DBM1Ac‐R strains as described in detail previously.^[^ ^8^, ^36^ ^]^ In brief, the DBM1Ac‐S and DBM1Ac‐R strains were kindly presented to our lab by Drs. Jianzhou Zhao and Anthony M. Shelton (Cornell University, USA) in 2003. The DBM1Ac‐S strain also referred to as Geneva 88, was obtained from an insecticide‐free cabbage research field at the New York State Experiment Station Robbins Farm in Geneva, New York, USA in 1988, and it had been kept in a Bt Cry toxin‐free environment since then. The DBM1Ac‐R, also referred to as Loxahatchee a, was a field‐evolved strain that was collected from commercial fields of kohlrabi in Loxahatchee, Florida, USA in 1992, it initially developed a 1641‐fold resistance to Javelin (Bt var. *kurstaki*) and was further subjected to additional selection using Cry1Ac‐expressing broccoli. Then, in the lab, the NIL‐R strain was established through six crossings between DBM1Ac‐S and DBM1Ac‐R, with selection using Cry1Ac protoxin in each generation to eliminate the genetic background differences in both strains. The resistance to Cry1Ac in NIL‐R was monogenic, incompletely recessive, and autosomal, and there were no apparent fitness costs associated with the resistance in the NIL‐R strain. Compared to the susceptible DBM1Ac‐S strain, the NIL‐R strain presents ≈5000‐fold resistance. The *P. xylostella* larvae of DBM1Ac‐S and NIL‐R strains were fed with Jing Feng No. 1 cabbage (*Brassica oleracea* var. *capitata*) at 25 °C with 65% relative humidity (RH) and a 16:8 (light: dark) photoperiod, and adults were supplied with a 10% honey/water solution. The Cry1Ac‐48 h strain was obtained from susceptible *P*. larvae in early third‐instar, which were treated with Jing Feng No. 1 cabbage coated with a Cry1Ac at an LC_50_ concentration of 1 mg L^−1^ for 48 h.

The *Spodoptera frugiperda* Sf9 cells (Invitrogen) were cultured in Sf‐900 II SFM (Gibco) insect cell culture medium supplemented with 50 mg L^−1^ Penicillin‐Streptomycin (Gibco). The cells were kept at 27 °C and regularly passaged every 4–5 days. The HEK293T cells (Thermo Fisher Scientific) were maintained in Dulbecco's Modified Eagle Medium (DMEM) medium (Gibco) supplemented with 10% fetal bovine serum (FBS) (Gibco) and 1% penicillin‐streptomycin (Gibco). The cells were cultured at 37 °C in 5% CO_2_ and regularly passaged every 3–4 days. The *Drosophila* S2 cells were maintained in SFX‐insect medium (HyClone) with 50 mg L^−1^ penicillin‐streptomycin (Gibco). The cells were cultured at 27 °C and passaged every 2–3 days.

### RNA Extraction and cDNA Synthesis

Fourth‐instar larvae of *P. xylostella* were used to dissect different tissue samples in ice‐cold insect Ringer's solution (0.5 mM KCl, 130 mM NaCl, 0.1 mM CaCl_2_). Total RNA was isolated using TRIzol reagent (Invitrogen). Then, the cDNA for qPCR detection of different tissues and development stages was prepared using the PrimeScript RT reagent Kit (TaKaRa), and the cDNA for gene cloning was synthesized using the PrimeScript II 1st strand cDNA Synthesis Kit (TaKaRa) according to the provided manuals.

### Dot Blot Analysis for RNA Modifications

Total RNA for dot blot assays was isolated from 30 midguts of fourth‐instar larvae of the DBM1Ac‐S or NIL‐R strain. The total RNA was isolated using TRIzol reagent (Invitrogen). The concentration of the RNA samples was detected using a Nanodrop 2000c (Thermo Fisher Scientific). Then, the RNA sample was divided into subgroups of 400, 200, and 100 ng µL^−1^. The RNA subgroups were denatured at 95 °C for 5 min and cooled on ice for 3 min. Subsequently, 2 µL samples were loaded onto Amersham Hybond‐N^+^ membranes (GE Healthcare), two membranes were used for each modification to allow detection of the modification level and total amount of input RNA. The membranes were UV cross–linked twice with 2000 joules each time. Then, one membrane was stained with 0.02% methylene blue (Solarbio), and another was blocked with Bløk‐CH buffer (Merck Millipore) for 3 h. The membrane was incubated with primary antibody, m^6^A (1:5000, Synaptic Systems), m^5^C (1:5000, Abcam), and m^1^A (1:5000, Abcam) overnight at 4 °C. The membrane was further incubated with a secondary antibody at room temperature for 30 min. The SuperSignal West Pico Chemiluminescent Substrate (Thermo Fisher Scientific) was used to visualize the dot blots, and the Tanon‐5200 Chemiluminescent Imaging System (Tanon) was used to capture images.

### HPLC‐MS/MS Analysis for RNA Modifications

Total RNA for high‐performance liquid chromatography coupled to tandem mass spectrometry (HPLC‐MS/MS) assays were isolated from 30 midguts of fourth‐instar *P. xylostella* larvae of the DBM1Ac‐S, Cry1Ac‐treated DBM1Ac‐S, and NIL‐R strains using the TRIzol reagent (Invitrogen). The concentrations of the RNA samples were determined using a Nanodrop 2000c (Thermo Fisher Scientific). Single nucleosides were generated from total RNA by a digestion buffer containing phosphodiesterase I (0.01 U) (Sigma Aldrich), nuclease P1 (1 U) (New England Biolabs), 2.5 mM zinc chloride (Sigma Aldrich) and 25 mM sodium acetate (Sigma Aldrich) at pH 6.8 for 2.5 h at 37 °C, followed by dephosphorylation with bacterial alkaline phosphatase (10 U) (Invitrogen) for 1 h at 37 °C. Nucleosides were separated on a Hypersil GOLD aQ reverse phase column (Thermo Scientific) and quantified with the nucleoside‐to‐base ion mass transitions of 258.1–126.1 for m^1^A and m^5^C, and 282.1–150.1 for m^6^A using HPLC‐MS/MS analysis on an Agilent 6490 Triple Quadrupole mass spectrometer.

### Bioinformatic Analysis

Gene exon‐intron analysis was performed using DNAMAN 9.0 (Lynnon BioSoft). The conserved domains of transmethylase proteins were analyzed using the Conserved Domain Database (CDD) of GenBank (https://www.ncbi.nlm.nih.gov/cdd/). The phylogenetic trees of transmethylase proteins were performed by MEGA 7.0 software (https://www.megasoftware.net/) with the neighbor‐joining (NJ) method following the p‐distance model, and 1000 bootstrap replicates. The m^6^A site of the *PxJHE* gene was predicted using SRAMP (http://www.cuilab.cn/sramp/).

### Cloning of the m^6^A Methyltransferase Subunit Genes and PxJHE Gene

The coding sequences (CDSs) of PxMettl3, PxMettl14, PxWTAP, PxSpenito, PxVirlizer, PxFlacc, PxHakai, and PxJHE genes in P. xylostella were obtained from the GenBank database (https://www.ncbi.nlm.nih.gov/) with the following accession numbers (PxMettl3, XM_048628151; PxMettl14, XM_011552978; PxWTAP, XM_011549585; PxSpenito, XM_011558003; PxVirlizer, XM_011558189; PxFlacc, XM_038120833; PxHakai, XM_011553442; PxJHE, XM_011558701). The CDS sequences of these genes were in silico corrected using the P. xylostella transcriptome database derived in this work. Gene‐specific primers for cloning the full‐length cDNA sequences of these genes were designed using the Primer Premier 5.0 software (Premier Biosoft). Ultra HiFidelity PCR Kit (TIANGEN) was used for PCR amplification. The PCR products were purified using the Monarch DNA Gel Extraction Kit (New England Biolabs), and the purified products were subcloned into the pEASY‐Blunt vector (TransGen) and sequenced. The full‐length cDNA sequences of PxMettl3, PxMettl14, PxWTAP, PxSpenito, PxVirlizer, PxFlacc, PxHakai, and PxJHE genes had been submitted to the GenBank database (accession nos. OQ291273‐OQ291280, respectively).

### qPCR Analysis

The gene‐specific primers of *PxMettl3*, *PxMettl14*, *PxWTAP*, *PxSpenito*, *PxVirlizer*, *PxFlacc*, *PxHakai*, and *PxJHE* gene used for real‐time quantitative PCR (qPCR) analysis were designed using the Primer Premier 5.0 software (Premier Biosoft) (Table S1, Supporting Information). The 2.5 × SYBR Green MasterMix Kit (TIANGEN) was used to detect the transcript levels of these genes using a QuantStudio 3 Real‐Time PCR System (Applied Biosystems), and the procedure involved initial denaturation at 94 °C for 3 min, followed by 40 cycles at 95 °C for 15 s, 60 °C for 30 s, and 72 °C for 30 s. The 2^−ΔΔCt^ method was used to calculate the relative expression of the target gene, and the relative expression of the target gene was normalized to the ribosomal protein L32 (*RPL32*) gene (GenBank accession no. AB180441) as before.^[^ ^37^ ^]^ Each sample was performed with three biological and four technical replicates.

### siRNA Knockdown

The specific siRNAs were designed in the gene‐specific region for knockdown of *PxMettl3*, *PxMettl14*, and *PxJHE*. The designed siRNA sequences were blasted against the GenBank database (https://www.ncbi.nlm.nih.gov/), two *P. xylostella* genome databases (DBM‐DB, http://116.62.11.144/DBM and Lepbase http://ensembl.lepbase.org/Plutella_xylostella_pacbiov1/) were used to confirm no specific hits to other genes. Then, the siRNAs were chemically synthesized by Shanghai GenePharma Co., Ltd. The gene‐specific siRNA sequences are listed below, a dTdT overhang was added at the 3′‐end of each sequence, and the C and U of the siRNAs were replaced by 2′‐methoxyl‐nucleotides, a scrambled siRNA was used as a negative control (siNC):

siMettl3: 5′‐GTGCGTTCAACACCAACAA‐3′

Sense: 5′‐GUGCGUUCAACACCAACAAdTdT‐3′

Antisense: 3′‐dTdTCACGCAAGUUGUGGUUGUU‐5′

siMettl14: 5′‐GATCCCAGAAACGGAAGAA‐3′

Sense: 5′‐GAUCCCAGAAACGGAAGAAdTdT‐3′

Antisense: 3′‐dTdTCUAGGGUCUUUGCCUUCUU‐5′

siJHE: 5′‐CGCGATACTTCCCAAACAA‐3′

Sense: 5′‐CGCGAUACUUCCCAAACAAdTdT‐3′

Antisense: 3′‐dTdTGCGCUAUGAAGGGUUUGUU‐5′

siNC: 5′‐TTCTCCGAACGTGTCACGT‐3′

Sense: 5′‐UUCUCCGAACGUGUCACGUdTdT‐3′

Antisense: 3′‐ dTdTAAGAGGCUUGCACAGUGCA‐5′

The synthesized siRNAs were dissolved in an injection buffer (Merck Millipore), and an equal volume of the Metafectene Pro transfer agent (Biontex) was added to promote siRNA transfection into cells. The mixture was injected into the newly molted third‐instar *P. xylostella* larvae with a sublethal dose of siRNA (25 µM, 70 nL) using a Nanoliter 2000 microinjection system (World Precision Instruments). Buffer and siNC were used as negative controls. The interference efficiency was tested at 48 h post‐injection using qPCR and western blot assays.

### Protein Extraction and Western Blot

Midguts from 30 fourth‐instar larvae of Bt‐susceptible, Bt Cry1Ac‐induced, or ‐resistant strains were dissected to detect the protein levels. The larvae were collected at 48 h post‐siRNA injection to detect the interference efficiencies. The midgut tissues or the whole larvae after siRNA injection were homogenized in cell lysis reagent (Sigma Aldrich) with protease inhibitor cocktail (Roche) and phosphatase inhibitor cocktail (Roche), and then the supernatant was collected after centrifugation. The protein concentration was quantified using the Bradford assay (Biomed). The protein was mixed with loading buffer (CWBIO) and separated using 10% SDS‐PAGE (CWBIO). Then, the separated proteins were transferred to a PVDF membrane (Merck Millipore), and the membrane was blocked using Bløk‐CH buffer (Merck Millipore). The membrane was incubated with primary antibody (METTL3, 1:5000, Proteintech; METTL14, 1:3000, AtaGenix) overnight at 4 °C, and then incubated with secondary antibody (1:5000, CWBIO) at room temperature for 1 h. The SuperSignal West Pico Chemiluminescent Substrate (Thermo Fisher Scientific) was used to visualize the bands, and the Tanon‐5200 Chemiluminescent Imaging System (Tanon) was used to capture images. ImageJ 1.51 software was used for densitometry.

### Toxin Preparation and Bioassay

Cry1Ac protoxin was isolated from Btk strain HD‐73, using methods of purification and quantification described in detail previously.^[^ ^38^ ^]^ Cry1Ac protoxin bioassays of all *P. xylostella* larvae were performed using a leaf‐dip method.^[^ ^39^ ^]^ Briefly, for Cry1Ac protoxin, after the interference of *PxMettl3*, *PxMettl14*, or *PxM3‐M14*, early third‐instar larvae microinjected with siPxMettl3, siPxMettl14, or siPxM3‐M14 were reared on fresh cabbage leaves soaked with 2,000 mg L^−1^ Cry1Ac protoxin. For bioassays after knockdown of *PxJHE*, early third‐instar larvae microinjected with siPxJHE were reared on fresh cabbage leaves soaked with 1 mg L^−1^ Cry1Ac protoxin. For each treatment of Cry1Ac protoxin, 40 early third‐instar were tested in each of the three biological replicates and the statistics on larval mortality were calculated at 72 h post‐treatment.

### Juvenile Hormone Extraction and Detection

JH extraction and detection were performed as described in detail previously.^[^ ^8b^ ^]^ Briefly, to determine the JH titer in resistant *P. xylostella* after siRNA knockdown, the samples were weighed and homogenized in glass homogenizers with 2 mL of ice‐cold methanol/ether (1:1, v/v), and 0.1 ng JH analog methoprene (Dr. Ehrenstorfer GmbH; 98.5%) was added as an internal standard. The homogenates were mixed by vortexing and centrifuged three times for 10 min at 4500 × *g* at 4 °C with 2 mL of *n*‐hexane to collect the hexane (upper) phase. The combined extracts were dried completely using a nitrogen blower at 20 °C and dissolved in a 500 µL mobile phase (70% methanol). Prior to chromatographic analysis, the final solution was filtered using a 0.22 µm membrane.

Mixed calibration standards were prepared using 70% methanol for JH II (SciTech; 78%) at concentrations of 0.01, 0.1, 0.5, 1, 2, 5, and 10 ng mL^−1^. 50 ng methoprene was added to each solution as an internal standard. Calibration curves were constructed by plotting the relative peak area ratios (the peak area of hormones/the peak area of their internal controls) against their concentrations. A weighted factor (1/y) least‐squares linear regression model was used to calculate the regression lines. The calibration curves of JH II were found to be linear over a range of concentrations with perfect regression correlation coefficients (*R* ^2^ = 1), which indicates that the hormone detection methods used were specific and accurate.

To accurately quantify the JH titer, the sample was subjected to chromatographic analysis using the ACQUITY UPLC I‐Class/Xevo TQ‐S micro System (Waters) equipped with an ACQUITY UPLC BEH C18 column (2.1 mm × 50 mm, 1.7 µm particle size). Chromatographic separation of JH was achieved with mobile phase A (methanol) and mobile phase B (water containing 0.1% formic acid). The gradient elution method was used to detect JH. The gradient started with 30% of component B, (water with 0.1% formic acid), for a duration of 2 min. The gradient was then decreased to 15% of component B over the next 2 min and maintained for another 2 min. Following this, the gradient was increased to 30% of component B over the next 2 min. The entire run lasted 9 min, including a 1 min equilibration step. The flow rate of the mobile phase remained constant at 0.2 mL min^−1^ with an injection volume of 10 µL, and the column temperature was kept at 40 °C. The system operation, data acquisition, and analysis were conducted using the MassLynx V4.1 software (Waters). Ten biologically independent samples with 3 larvae per sample were run.

### Fitness Costs Analysis

The biological parameters of fitness costs were pupation rate, eclosion rate, pupal weight, pupal duration, adult longevity, oviposition duration, fecundity, and hatchability. Newly molted third‐instar larvae injected buffer and siNC were used as negative controls. All injected larvae were fed on fresh cabbage leaves. For pupation rate, eclosion rate, and hatchability, three replicates were performed, and ten larvae per replicate. For pupal weight, pupal duration, adult longevity, oviposition duration, and fecundity, 30 replicates were performed, and one larva per replicate. The statistical significance of differences in these biological parameters was assessed using one‐way ANOVA with Tukey's test (overall significance level = 0.05) between negative controls and treated groups.

### MeRIP‐seq

For MeRIP‐seq analysis, 40 midguts from fourth‐instar larvae were used in each biological replicate, and each strain included three biological replicates. Total RNA was isolated using TRIzol reagent (Invitrogen), and the purity and concentration of the total RNA were detected by a NanoDrop ND‐1000 spectrophotometer (Thermo Fisher Scientific). RNA integrity was assessed by the Agilent 2100 bioanalyzer with RNA integrity number (RIN) > 7.0 and further confirmed by electrophoresis with denaturing agarose gel. Poly (A) RNA was extracted from 50 µg total RNA with Dynabeads Oligo (dT) 25–61005 (Thermo Fisher Scientific) and the extraction was performed in two rounds. The poly (A) RNA was fragmented using the NEBNext Magnesium RNA Fragmentation Module (New England Biolabs) at 86 °C for 7 min. Then, a portion of fragmented RNA was used to generate input control and RNA‐seq libraries. The input control is required for determining signal enrichment in the immunoprecipitated sample. Another part of the fragmented RNAs was incubated with the m^6^A‐specific antibody (Synaptic Systems) in IP buffer (750 mM NaCl, 50 mM Tris‐HCl, and 0.5% Igepal CA‐630) at 4 °C for 2 h. Then, the precipitated RNA was reverse‐transcribed to generate cDNA using the SuperScript II Reverse Transcriptase (Invitrogen), and the obtained cDNA was used to synthesize U‐labeled second‐stranded DNAs with *Escherichia coli* DNA, RNase H (New England Biolabs), dUTP Solution (Thermo Fisher Scientific), and polymerase I (New England Biolabs). The indexed adapters were prepared using the blunt ends with an A‐base. The A‐tailed fragmented DNA was ligated to the adapter with a T‐base, and the AMPureXP beads were used to select the size of the index adapters. The U‐labeled second‐stranded DNAs were treated with heat‐labile UDG enzyme (New England Biolabs), the ligated fragments were amplified using PCR with the following conditions: initial denaturation at 95 °C for 3 min; denaturation at 98 °C for 15 s with 8 cycles, annealing at 60 °C for 15 s, and extension at 72 °C for 30 s; and then final extension at 72 °C for 5 min. For the final cDNA library, the size of the insert fragments was 300 ± 50 bp. Finally, 2 × 150 bp paired‐end sequencing (PE150) was performed on an Illumina Novaseq 6000 platform (LC‐Bio Technology) following the vendor's recommended protocol.

### MeRIP‐seq Data Analysis

For MeRIP‐seq data analysis, reads that contained low‐quality bases, adaptor contamination, or undetermined bases were removed using fastp (https://github.com/OpenGene/fastp) with default parameters. The reads were mapped to the reference genome of *P. xylostella* deposited in GenBank database (https://www.ncbi.nlm.nih.gov/bioproject/PRJEB34571/) using HISAT2 (http://www.ccb.jhu.edu/software/hisat/). BAM files obtained from input and IP samples, R package exomePeak (https://bioconductor.org/packages/3.9/bioc/html/exomePeak.html) were used for peak calling and differential analyses. The resulting bigWig files identified m^6^A peaks that were visualized with the Integrative Genomics Viewer (IGV) software (http://www.broadinstitute.org/igv/). De novo and known motif discovery were performed by MEME (https://meme.nbcr.net/) and HOMER (https://www.homerenergy.com/products/pro/index.html), and then the motif with respect to peak summit was located. R package ChIPseeker (http://www.bioconductor.org/packages/release/bioc/html/ChIPseeker.html) was used to annotate the called peaks by intersection with gene architecture. The expression of all mRNAs from input libraries was performed using StringTie (http://ccb.jhu.edu/software/stringtie) by calculating FPKM (fragments per kilobase of transcript per million mapped reads). R package edgeR (http://bioconductor.org) was used to select the differentially expressed mRNAs.

### Ectopic Expression and Protein Purification

The full‐length cDNA sequence of the *PxJHE* gene with His‐tag was cloned into the pFastBac1 expression vector (Invitrogen). 2 mL Sf9 cells (0.5 × 10^6^ mL^−1^) were seeded into a 6‐well plate and incubated at 27 °C in an incubator for cell adhesion. 5 µg pFastBac1 vector was transfected into adherent Sf9 cells using transfection reagent (Thermo Fisher Scientific), and Sf9 cells were cultured in an incubator at 27 °C until the cells were transfected. The cells were centrifuged to obtain the P1 generation virus in the supernatant. The supernatant was added to 30 mL Sf9 cells (2.0 × 10^6^ mL^−1^), and the cells were cultured in a shaker at 120 rpm at 27 °C to obtain the P2 generation virus for expanding the virus population. The P2 generation virus was obtained by centrifuging. The P2 generation virus was added to 1 L Sf9 cells (2.0 × 10^6^ mL^−1^), and the cells were cultured in shaker at 120 rpm at 27 °C to amplify protein expression. The sample was centrifuged to obtain supernatant, and the proteins were purified using Ni‐column (Yuanye Biotech), and the protein purity was verified with SDS‐PAGE followed by Coomassie staining and western blot (Figure S4, Supporting Information).

### Metabolic Experiments

Metabolic analysis of the PxJHE protein was investigated by UPLC‐MS/MS analysis using the ACQUITY UPLC I‐Class/Xevo TQ‐S micro System (Waters) equipped with an ACQUITY UPLC BEH C18 column (2.1 mm × 50 mm, 1.7 µm particle size), and JH II standard (SciTech; 78%) were used as metabolic substrate. 0.18 µg recombinant PxJHE protein was added to 50 µL reaction volume containing 20 µg JH II. After incubation at 30 °C for 5 min, the reaction was stopped by adding 50 µL methanol. Subsequently, 100 µL of the sample was transferred to a brown loading bottle, and the product was further detected by the ACQUITY UPLC I‐Class/Xevo TQ‐S micro System (Waters). The column temperature was set to 40 °C. A methanol/water (0.1% formic acid) gradient elution was carried out at a flow rate of 0.2 mL min^−1^ as follows: 0–2 min (70% methanol), 2–4 min (85% methanol), maintained for another 2 min, 6–8 min (70% methanol) with an injection volume of 10 µL. The entire run lasted 9 min, including a 1 min equilibration step. Data acquisition and analysis were performed using the MassLynx V4.1 software (Waters) as mentioned above.

### m^6^A‐IP‐qPCR

The validation of the m^6^A site of *PxJHE* and detection of the m^6^A level of *PxJHE* in the midgut of DBM1Ac‐S, NIL‐R, buffer‐treated, siNC‐treated, siPxMettl3‐treated, siPxMettl14‐treated, and siPxM3‐M14‐treated strains were performed using the Magna MeRIP m^6^A Kit (Merck Millipore). 600 µg total RNA from the midgut of fourth‐instar larvae was extracted using TRIzol reagent (Invitrogen). Total RNA was fragmented using a fragmenting reagent (supplied with kit) and taking out 10% from fragmented RNAs as input. Anti‐m^6^A antibody (supplied with kit) or control IgG antibody (supplied with kit) was incubated with protein A/G agarose beads (supplied with kit) at room temperature for 30 min, followed by incubation with the fragmented RNAs in IP buffer (supplied with kit) at 4 °C for 2 h. The samples were eluted twice with the eluent for 1 h each time at 4 °C. The eluted RNA was precipitated with one‐tenth volume of 3 M sodium acetate (pH 5.2) (Thermo Fisher Scientific) and 2.5 volumes of absolute ethanol (Innochem), and the samples were reverse transcribed using the PrimeScript RT reagent Kit (TaKaRa) according to the manuals. The m^6^A level of the *PxJHE* gene was tested by qPCR using site‐specific primers (Table S1, Supporting Information), and related enrichment of m^6^A in the special sample was calculated by normalizing to input.

### Dual‐Luciferase Reporter Assays

The dual luciferase assays were used to verify the function of the m^6^A site of *PxJHE* gene in HKT‐293T cells as described elsewhere.^[^ ^40^ ^]^ The pmirGLO luciferase expression vector (Promega) with a firefly luciferase (F‐luc) and a Renilla luciferase (R‐luc) was used to construct the fluorescent reporter plasmid as described elsewhere.^[^ ^41^ ^]^ Wild‐type PxJHE fragment (*PxJHE*‐wt) contained CDS and 3′‐UTR of *PxJHE* and mut‐type PxJHE fragment (*PxJHE*‐mut) was made by replacing the adenosine bases (A) with cytosine (C). The fragment of *PxJHE*‐wt and *PxJHE*‐mut was attached to the pmirGLO vector by In‐Fusion HD Cloning Kit (TaKaRa) according to the instructions. The HKT‐293T cells were seeded in a 24‐well plate using DMEM medium with 10% FBS and 1% antibiotics, and cells were cultured in a CO_2_ incubator at 37 °C. Transfection experiments were performed when the cell density was ≈ 80%. 200, 400, 600, and 800 ng plasmid of *PxJHE*‐wt or *PxJHE*‐mut were transfected into HKT‐293T cells cultured in DMEM medium with 10% FBS using Lipofectamine 3000 (Invitrogen). After 48 h, the cells were lysed, and the fluorescence activity was detected using the Dual‐Glo Luciferase Assay System (Promega). The effect of the m^6^A site on *PxJHE* mRNA was evaluated by firefly luciferase activity, and firefly luciferase activity was normalized to Renilla luciferase activity.

### mRNA Stability Assay

The mRNA stability assay was performed using the *Drosophila* expression vector pAc5.1/V5‐His B (Invitrogen). The wild‐type and mut‐type *PxJHE* fragments containing the CDS and 3′‐UTR were prepared as the above dual‐luciferase reporter assays. The fragment of *PxJHE*‐wt and *PxJHE*‐mut was attached to the pAc5.1/V5‐His B vector by the In‐Fusion HD Cloning Kit (TaKaRa) according to the instructions. The *Drosophila* S2 cells were seeded in a 12‐well plate with SFX‐insect medium (HyClone). 600 ng *PxJHE*‐wt or *PxJHE*‐mut plasmid was transfected into S2 cells using Lipofectamine 2000 (Invitrogen). After 24 h, 10 mg L^−1^ actinomycin D (Sigma) was added. After 30 min of incubation, cells from 3 wells were collected and considered as time zero controls, subsequently, three wells were harvested every 2 h with each well of cells acting as a biological replicate. Total RNA was isolated using TRIzol reagent (Invitrogen), the cDNA for qPCR detection of the expression level of the *PxJHE* transcript was prepared using the PrimeScript RT reagent Kit (TaKaRa), and the primer was the same as that used for the qPCR analysis of *PxJHE* as mentioned above.

### RIP‐qPCR

A Magna Nuclear RIP (Cross‐Linked) Nuclear RNA‐Binding Protein Immunoprecipitation Kit (Merck Millipore) was used to perform the RNA immunoprecipitation. Sixty‐fourth‐instar larvae midguts were washed twice using ice‐cold PBS buffer solution (Thermo Fisher Scientific). To increase cross‐linking efficiency, the tissue was cut into pieces with sterilized scissors. Then, the sample was centrifuged for 5 min at 800 × *g* at 4 °C. The samples after removal of the supernatant were cross–linked by 37% formaldehyde. After incubation at room temperature for 10 min, 10 × glycine (supplied with kit) was added to quench excess formaldehyde. The sample was centrifuged for 5 min at 800 × *g* at 4 °C to remove the supernatant and washed twice with ice‐cold PBS buffer solution (Thermo Fisher Scientific). Samples were homogenized using a pre‐cooled homogenizer. The homogenized samples were centrifuged for 5 min at 800 × *g* at 4 °C. The samples were lysed to release the nucleus using the Nuclei Isolation Buffer (supplied with kit) containing 200 × Protease Inhibitor Cocktail III and RNase inhibitor (supplied with kit), and the RIP Cross‐linked Lysis Buffer (supplied with kit) containing 200 × Protease Inhibitor Cocktail III and RNase inhibitor (supplied with kit) was used to release cross‐linked proteins/RNA. Cross‐linked DNA was sheared to 200–1,000 bp using sonication. The sonication was performed at 40 w for 7 min, using 3 s pulses with 10 s intervals between pulses. Tubes were kept in an ice‐water mixture at all times. After centrifugation of the sonicated lysate, 5% of the supernatant was removed as input. The supernatant was subjected to Magna ChIP Protein A/G Magnetic Beads (supplied with kit) coupled with either 5 µg METTL3 antibody (Proteintech), METTL14 antibody (AtaGenix), or mouse IgG antibody (Merck Millipore) and rotated overnight at 4 °C. Protein/RNA complexes were incubated with Nuclear RIP Elution buffer (supplied with kit) containing 180 µg of proteinase K to digest protein. The RNA of input, IP, and IgG were precipitated with one‐tenth volume of 3 M sodium acetate (pH 5.2) (Thermo Fisher Scientific) and 2.5 volumes of absolute ethanol (Innochem), and the samples were reverse transcribed using the PrimeScript RT Reagent Kit (TaKaRa) according to the manuals. The level of METTL3 or METTL14 binding to *PxJHE* mRNA was tested by qPCR, and related enrichment of *PxJHE* mRNA was calculated by normalizing to input.

### CRISPR/Cas9 Experiment

CRISPR/Cas9‐mediated homology direct repair (HDR) pathway was implemented to replace the m^6^A site with a C base from the 3′‐UTR region of the *PxJHE* gene in Cry1Ac‐resistant NIL‐R strain. An optimal single guide RNA (sgRNA) was designed using the powerful CRISPR RGEN tool Cas‐Designer (http://www.rgenome.net/casdesigner/) according to the principle of 5′‐N20NGG‐3′ in the 3′‐UTR region of the *PxJHE* gene in NIL‐R strain, meanwhile, a 90‐nt single‐stranded oligodeoxynucleotide (ssODN) with the precise m^6^A replacement and two homology arms flanking the CRISPR target site was designed in vitro as a donor DNA to repair double‐stranded breaks (DSB) (Table S3, Supporting Information). The potential off‐target effect of the sgRNA sequence was eliminated by searching two *P. xylostella* genome databases (DBM‐DB, http://116.62.11.144/DBM and Lepbase http://ensembl.lepbase.org/Plutella_xylostella_pacbiov1/), the GenBank database (https://www.ncbi.nlm.nih.gov/), and by using the CRISPR RGEN Cas‐OFFinder tool (http://www.rgenome.net/casoffinder/). The DNA template for sgRNA synthesis by a fused PCR has been described in detail elsewhere.^[^ ^8d^ ^]^ The high yield of sgRNA was obtained by in vitro MEGAshortscript Transcription Kit (Ambion) and purified by the MEGAclear Kit (Ambion) following the instructions. The mixture of sgRNA (150 ng/µl), Cas9 protein (100 ng µL^−1^), and ssODN (1 mg mL^−1^) was microinjected into fresh eggs from the NIL‐R strain using the FemtoJet 4i and InjectMan 4 Microinjection System (Eppendorf). To assess the CRISPR/Cas9‐induced site‐specific mutation, we extracted the gDNA from the tiny exuviates of final fourth‐instar *P. xylostella* larvae and designed specific primers for amplifying the gDNA fragments flanking sgRNA target sites. Individual larvae with the site‐specific mutation were identified by direct sequencing of PCR products. Finally, the stable homozygous mutant strain (m^6^A‐KO) was constructed by efficient mutation screening and optimized germline transformation strategies (Figure S6B and Table S4, Supporting Information).

### Statistical Analysis

All experiments were performed with at least three independent biological replicates in the lab. The pre‐processing of data and sample size (*n*) for each statistical analysis are listed in the figure legends. Data are presented as mean values ± SEM. Statistical analysis was performed using the IBM SPSS Statistics 23.0 software. When comparing the means of more than two groups, one‐way ANOVA with Tukey's test was used for comparison. A *p*‐value less than 0.05 was considered statistically significant: *p < 0.05, **p < 0.01, ***p < 0.001.

## Conflict of Interest

The authors declare no conflict of interest.

## Author Contributions

Z.G., Y.B., X.Z., L.G., and L.Z. contributed equally to this work. Z.G., Y.B., N.C., and Y.Z. designed the research. Z.G., Y.B., X.Z., L.G., L.Z., D.S., K.S., X.X., X.Y., W.X., S.W., and Q.W. performed the experiments. Z.G., Y.B., X.Z., L.G., and N.C. analyzed the data. Z.G., Y.B., N.C., X.Z. and Y.Z. wrote and revised the manuscript.

## Supporting information

Supporting Information

## Data Availability

The data that support the findings of this study are available from the corresponding author upon reasonable request.
