# Supplementary material for: RNA m6A Methylation Suppresses Insect Juvenile Hormone Degradation to Minimize Fitness Costs in Response to A Pathogenic Attack
Source: Adv Sci (Weinh). 2023 Dec 12;11(6):2307650. doi: 10.1002/advs.202307650 (PMC10853702; doi:10.1002/advs.202307650)
Supplement: Supplementary file 1 — Supporting Information [file ADVS-11-2307650-s001.pdf]

ADVANCED SCIENCE
Open Access

Supporting Information

for *Adv. Sci.*, DOI 10.1002/advs.202307650

RNA m^{6}A Methylation Suppresses Insect Juvenile Hormone Degradation to Minimize Fitness Costs in Response to A Pathogenic Attack

Zhaojiang Guo, Yang Bai, Xinyi Zhang, Le Guo, Liuhong Zhu, Dan Sun, Kaiyue Sun, Xudan Xu, Xin Yang, Wen Xie, Shaoli Wang, Qingjun Wu, Neil Crickmore, Xuguo Zhou and Youjun Zhang**

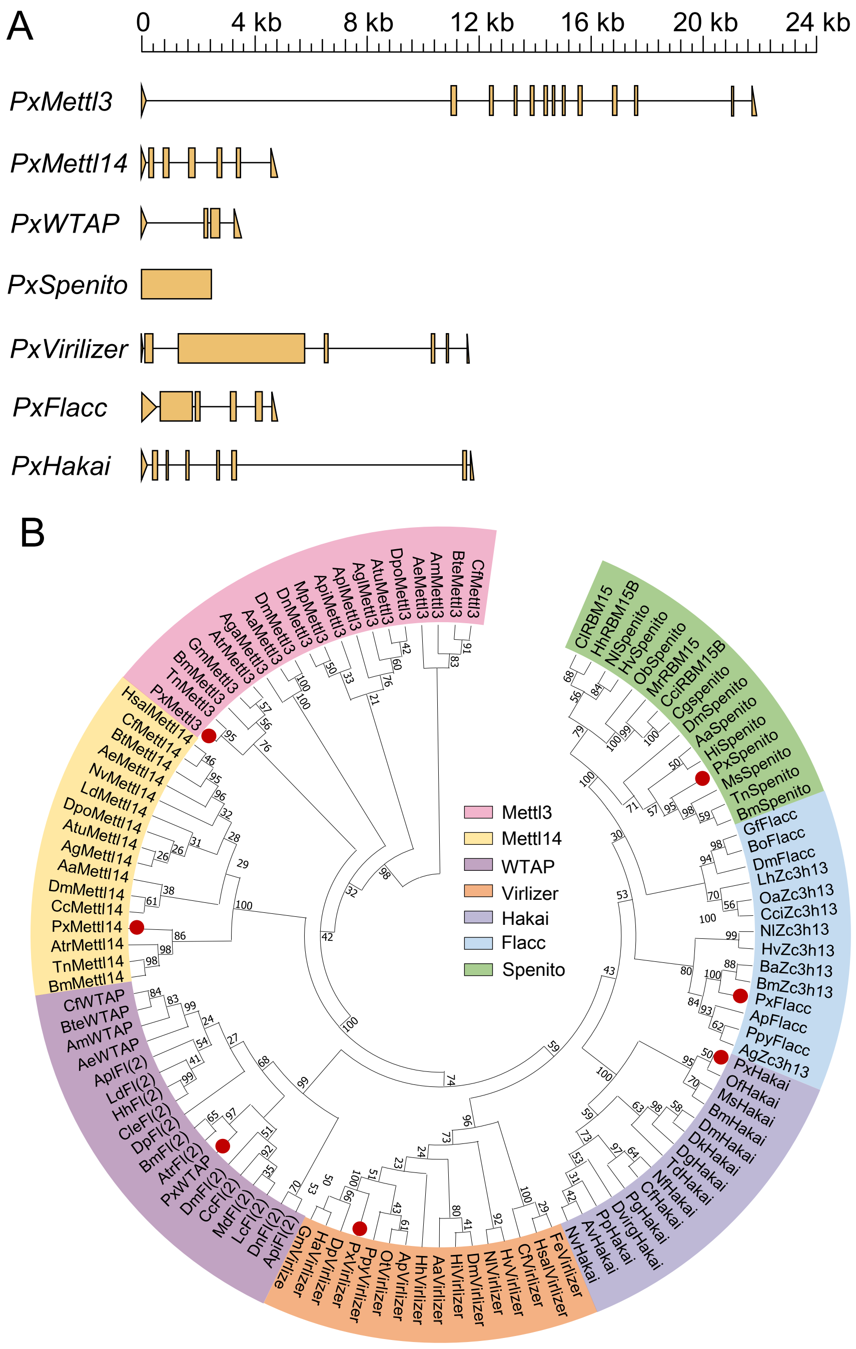

Figure S1. Characterization of *P. xylostella* m^{6}A methyltransferase subunit genes. A) Genomic structure analysis of the m^{6}A methyltransferase subunit genes. The genomic structures of these genes are drawn to scale. Exons are displayed as boxes and triangles, and the spaces between two exons denote the introns. B) Phylogenetic analysis of the m^{6}A methyltransferase subunit proteins from different species. The full-length amino acid sequences of the m^{6}A methyltransferase subunit proteins of *P.*

xylostella are highlighted in red solid circles. Full-length amino acid sequences of all the m^{6}A methyltransferase subunit proteins from other species were retrieved from the GenBank database as listed in Table S2.

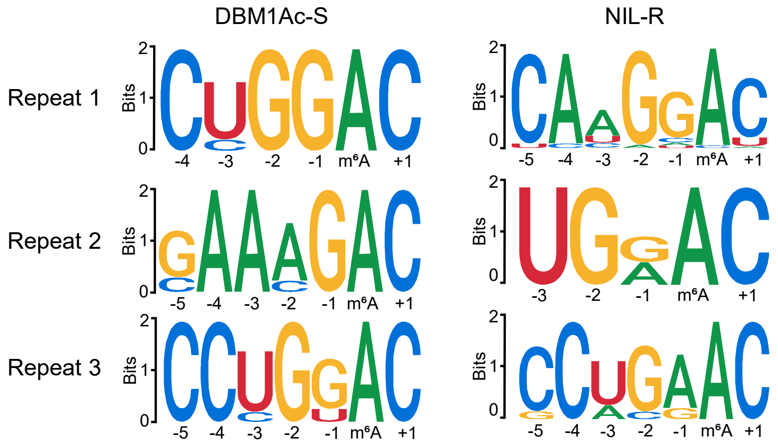

Figure S2. Sequence motifs derived from MeRIP-seq analysis. Sequence motifs were determined from the top 1,000 most significant m^{6}A peaks from the 3 biological repeats of both susceptible DBM1Ac-S and resistant NIL-R strains.

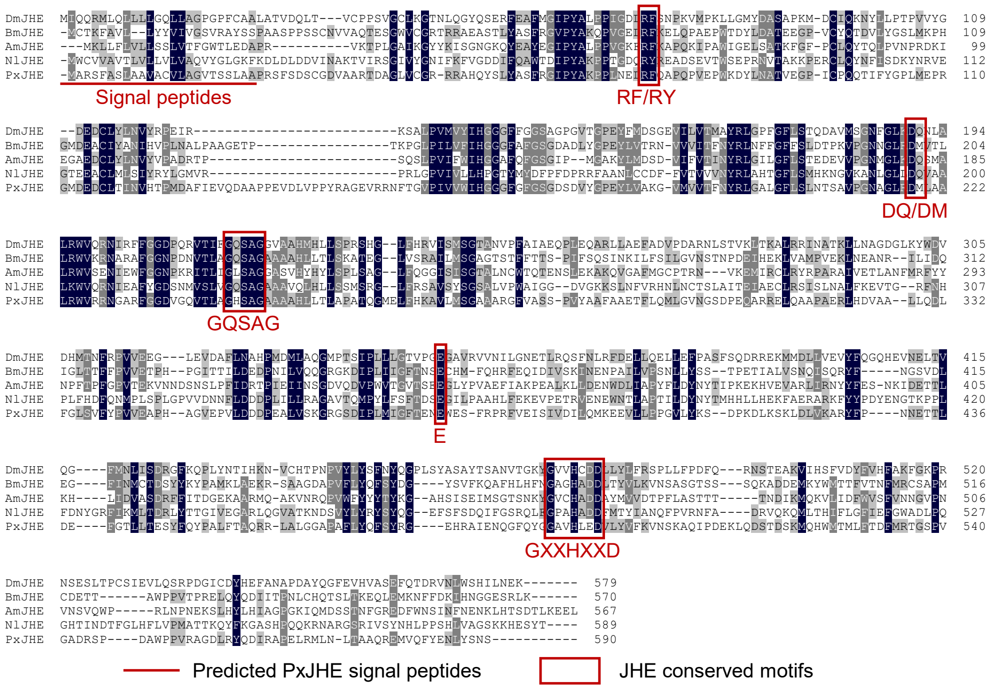

Figure S3. Multiple sequence alignment of JHE from different insects. Juvenile hormone esterase harbors five relatively conserved catalytic sites of JH (indicated by red boxes) and a signal peptide (indicated by red line). Full-length amino acid

sequences of the PxJHE from other insects were retrieved from the GenBank database (Dm, *Drosophila melanogaster*, AAK07833; Bm, *Bombyx mori*, AAL55240; Am, *Apis mellifera*, NP_001011563; Nl, *Nilaparvata lugens*, ACB14344).

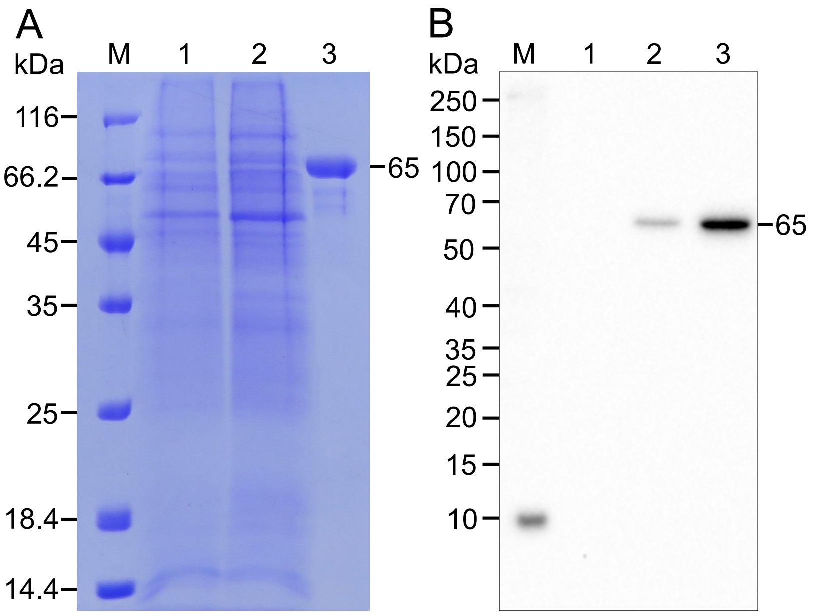

Figure S4. Heterologous expression of PxJHE protein in Sf9 cells. A) The expression and purification of PxJHE protein in Sf9 cells detected by SDS-PAGE. B) Protein detection by western blot. Lane M, protein marker; lane 1, the supernatant of Sf9 cells transfected with an empty vector (control); lane 2, the supernatant of Sf9 cells transfected with PxJHE expressing construct; lane 3, purified PxJHE protein.

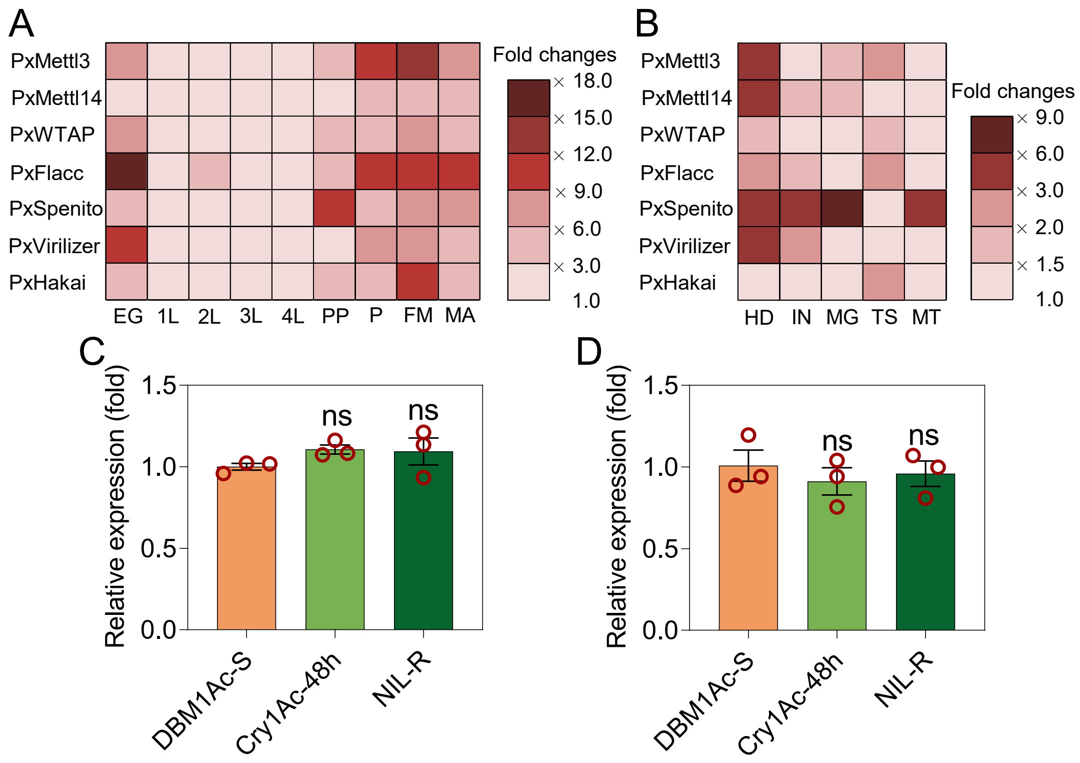

Figure S5. Expression analysis of the m^{6}A transmethylase subunit genes at different development stages and in different tissues of *P. xylostella***.** A and B) Heat map showing the relative expression of m^{6}A transmethylase subunit genes at different development stages (A) and in different tissues (B) of susceptible DBM1Ac-S strain as detected by qPCR. For different tissues (HD, head; IN, integument; MG, midgut; TS, testis; MT, Malpighian tubules) and developmental stages (EG, eggs; L1-4, first- to fourth-instar larvae; PP, prepupae; P, pupae; MA and FA, male and female adults). The colors of each rectangle represent the relative transcription level of each gene expressed as mean fold change compared to the tissue or developmental stage with the lowest expression level. The color keys from light pink to deep red represent the relative expression levels from low to high. C and D) The relative expression levels of the *PxMettl3* (C) and *PxMettl14* (D) genes in the whole body of Bt susceptible, Bt Cry1Ac-treated, and Bt resistant *P. xylostella* strains. The relative expression levels of the *PxMettl3* and *PxMettl14* genes have been normalized to the

expression level of the internal control *RPL32* gene and the expression level of the DBM1Ac-S strain was set to 1.0.

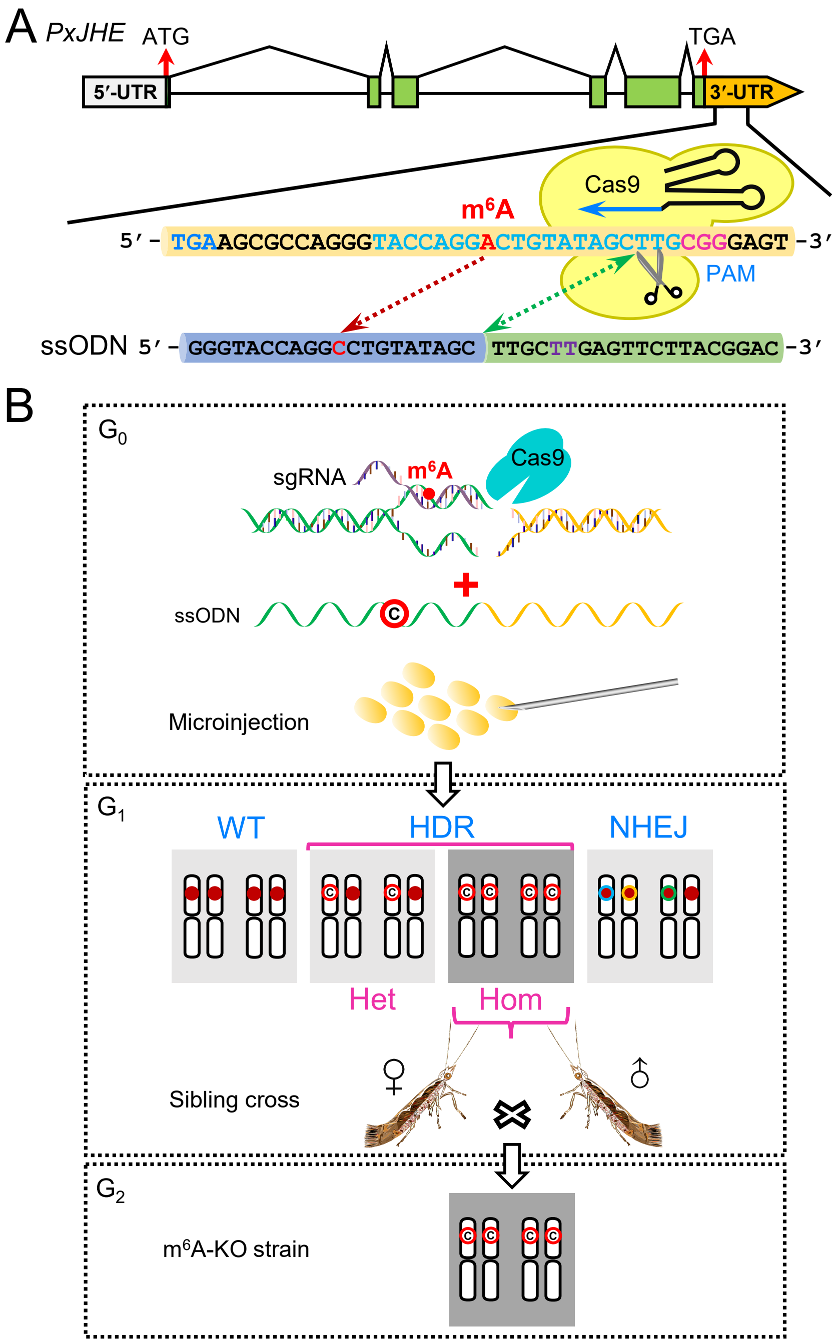

Figure S6. CRISPR/Cas9-mediated m^{6}A specific mutation in *P. xylostella*. A) The genomic structure of the *PxJHE* gene and the m^{6}A site-specific mutation mediated by the CRISPR/Cas9 genome editing system. The optimal sgRNA guides Cas9 protein to recognize the PAM site (NGG) and cleave the 3′-UTR region of the

PxJHE to create double-strand break (DSB), and then a ssODN template was designed to repair DSB by homology-directed repair (HDR) pathway. B) Diagram of the crossing strategy used to obtain homozygous m^6A knockout *P. xylostella* strain. A mixture of m^6A site-specific sgRNA, Cas9 protein, and ssODN was microinjected into fresh embryos from the NIL-R strain. The homozygotes with precise m^6A replacement in G_0 progeny were sib-crossed with each other to produce G_1 progeny. The homozygous G_1 individuals with m^6A site-specific mutation identified by direct sequencing of PCR products were sib-mated to generate G_2 progeny. From the G_0 adults, 50 male and 50 female moths were randomly selected to sibling cross to generate G_1 progeny, and 144 pupae were obtained for genotyping. A non-destructive method was used to extract the gDNA from exuviates of the final fourth-instar *P. xylostella* larvae, subsequently, the PCR products surrounding target sites were sequenced to determine the exact mutation types in G_1. Ultimately, 25 homozygous (11 males and 14 females) with precise m^6A replacement in G_1 progeny were sib-crossed with each other to establish a homozygous m^6A site-specific mutation *P. xylostella* strain named m^6A-KO. For these genotypes, white columns represent autosomes, the solid red circle indicates the m^6A site, and the hollow circles with different colors outside the red circles denote different mutation types produced by the CRISPR/Cas9-mediated non-homologous end joining (NHEJ) pathway. The letter C in the circle represents the m^6A site replaced with a C.

Table S1. Primers used in this study

Purpose	Gene	Primer name	Primer sequence (5′-3′)	PCR product (bp)	Positions
Full-length cDNA cloning	PxMettl3	Mettl3-F	TCAACGTTGACGAAGCTAAAGC	1813	-60/+1753
		Mettl3-R	ACACTTGTTACACTCGTCTCATCA		
	PxMettl14	Mettl14-F	TGGATTCCTCAAATTGACAAACAA	1256	-73/+1183
		Mettl14-R	GGCTGATGGAATTTAGCATCATT		
	PxWTAP	WTAP-F	AAATGGCGTCTGTGTCTGTG	1387	-164/+1223
		WTAP-R	ACATGGTCCACTGTTGTGCA		
	PxSpenito	Spenito-F	GTGCCGTGTCGTTAGTGACT	2732	-66/+2666
		Spenito-R	TGTTCACGCACTTACACTACAC		
	PxVirilizer	Virilizer-F1	TGGTTCTGGGCTTTTTGTGT	4043	-95/+4248
		Virilizer-R1	CAGCCTTCGCTCGTTGAATG		
		Virilizer-F2	GCTGTCAGAGCGCTTTTAGC	1904	+4003/+5906
		Virilizer-R2	AGGTGTTATACATTGGAGTAGGATGT		
	PxFlacc	Flacc-F	TGCCGGTGCAAAATTGTGAA	3156	-66/+3090
		Flacc-R	AAGGCATCGAACCGTTCCTT		
	PxHakai	Hakai-F	AGGACCAAGCTATCTTCTGTTTGT	1197	-26/+1171
		Hakai-R	GGGGTATAAATAGCGGCGCT		
	PxJHE	JHE-F	GCCCGACGACTGTTCTTTAT	2590	-41/+2549
		JHE-R	CGCAACGGTGGGTTTATGAC		
qPCR analysis	PxMettl3	qMettl3-F	ACGGCATCATCGAGAGACTG	163	+1523/+1685
		qMettl3-R	CCATCGGGGTAGCGTTTCTT		
	PxMettl14	qMettl14-F	GACGGAGAAGAATCCACCTGT	95	+890/+984
		qMettl14-R	CGCGTTGAAGTTGGAGTTGG		
	PxWTAP	qWTAP-F	TGCTTGTTCGAAGGCTAGCA	210	+2412/+2571
		qWTAP-R	ACTTCCACGCGTTGAGTTCA		
	PxSpenito	qSpenito-F	CGCATCTTTGGCCGTTATGG	137	+946/+1082

		qSpenito-R	TGCCCAGAGAGCTCTACCTT		
	PxVirilizer	qVirilizer-F	GTTTCCGGTAGGGTCGGTC	133	+3959/+4091
		qVirilizer-R	CCAAGGTCCTCTTCGGATCG		
	PxFlacc	qFlacc-F	GCGACGACCCCGATGATATAC	101	+2183/+2283
		qFlacc-R	ATTTGGTGCGCTTTCTTCAGC		
	PxHakai	qHakai-F	CTCAGGAAGCGGACGATACG	132	+143/+274
		qHakai-R	GACCTCGCTGCAATGTTGTG		
	PxJHE	qJHE-F	CAGAGAACGAGTGGGAGTCG	67	+1127/+1193
		qJHE-R	ATTTGGGAAGTATCGCGCCT		
	PxRPL32	qL32-F	CCAATTTACCGCCCTACC	120	—
		qL32-R	TACCCTGTTGTCAATACCTCT		
m^6A-IP-qPCR	Positive	P-JHE-m^6A-F	CGAGAACCTCTACAGCAACA	83	+1749/+1831
		P-JHE-m^6A-R	GTTTTCAATCGGTCCGTAAG		
	Negative	N-JHE-m^6A-F	GCCCGACGACTGTTCTTTAT	70	-41/+29
		N-JHE-m^6A-R	GCGGCGAGAGAAGCGAAACT		
pmirGLO recombinant	PxJHE	P-JHE-F	GCTCGCTAGCCTCGAGCCCGACGACTGTTCTTTATTTGAAA	2468	-41/+2427
		P-JHE-R	ATGCCTGCAGGTCGAATTAAACAAGAATGAGCCAT		
Mutation at m^6A site	PxJHE	M-JHE-F	ACCAGGCCTGTATAGCTTGCGGGAG	—	1791
		M-JHE-R	TACAGGCCTGGTACCCTGACGCTT		

Table S2. List of the m^6A methyltransferase subunit proteins from different insects as used in the phylogenetic analysis

Genes	Insect orders	Species	Name	Source	Gene ID	Size (aa)	Sequence quality
Mettl3	Lepidoptera	*Amyelois transitella*	AtrMettl3	GenBank	XP_013191837	563	Complete
		Bombyx mandarina	BmMettl3	GenBank	XP_028032854	569	Complete
		Galleria mellonella	HaMettl3	GenBank	XP_026750524	568	Complete
		Plutella xylostella	PxMettl3	GenBank	OQ291273	575	Complete
		Trichoplusia ni	TnMettl3	GenBank	XP_026728190	561	Complete
	Coleoptera	*Anoplophora glabripennis*	AglMettl3	GenBank	XP_018561753	574	Complete
		Agrilus planipennis	AplMettl3	GenBank	XP_018327837	561	Complete
		Aethina tumida	AtuMettl3	GenBank	XP_019866496	562	Complete
		Dendroctonus ponderosae	DpoMettl3	GenBank	XP_019772519	545	Complete
	Diptera	*Aedes aegypti*	AaMettl3	GenBank	XP_001662288	595	Complete
		Anopheles gambiae	AgaMettl3	GenBank	XP_312015	649	Complete
		Drosophila melanogaster	DmMettl3	GenBank	NP_651204	608	Complete
	Hymenoptera	*Acromyrmex echinatior*	AeMettl3	GenBank	XP_011057769	548	Complete
		Apis mellifera	AmMettl3	GenBank	XP_016767532	556	Complete
		Bombus terrestri	BteMettl3	GenBank	XP_012165914	563	Complete
		Camponotus floridanus	CfMettl3	GenBank	XP_011253998	548	Complete
	Hemiptera	*Acyrthosiphon pisum*	ApMettl3	GenBank	XP_001945512	550	Complete
		Diuraphis noxia	DnMettl3	GenBank	XP_015376446	584	Complete
		Myzus persicae	MpMettl3	GenBank	XP_022173579	550	Complete
Mettl14	Lepidoptera	*Amyelois transitella*	AtrMettl14	GenBank	XP_013195550	381	Complete
		Bombyx mori	BmMettl14	GenBank	XP_004924405	381	Complete
		Plutella xylostella	PxMettl14	GenBank	OQ291274	377	Complete
		Trichoplusia ni	TnMettl14	GenBank	XP_026733132	382	Complete
		Papilio polytes	PpMettl14	GenBank	XP_013148094	376	Complete
	Coleoptera	*Anoplophora glabripennis*	AglMettl14	GenBank	XP_018561874	393	Complete

		Aethina tumida	AtuMettl14	GenBank	XP_019868745	393	Complete
		Dendroctonus ponderosae	DpoMettl14	GenBank	XP_019766864	393	Complete
		Leptinotarsa decemlineata	LdMettl14	GenBank	XP_023029362	391	Complete
		Nicrophorus vespilloides	NvMettl14	GenBank	XP_017773393	413	Complete
	Diptera	*Aedes aegypti*	AaMettl14	GenBank	XP_021702143	391	Complete
		Ceratitis capitata	CcMettl14	GenBank	XP_004531408	406	Complete
		Drosophila melanogaster	DmMettl14	GenBank	NP_609205	397	Complete
	Hymenoptera	*Acromyrmex echinatior*	AeMettl14	GenBank	XP_011059005	392	Complete
		Bombus terrestri	BtMettl14	GenBank	XP_003402816	390	Complete
		Camponotus floridanus	CfMettl14	GenBank	XP_025264258	390	Complete
		Harpegnathos saltator	HaslMettl14	GenBank	XP_019695918	397	Complete
WTAP	Lepidoptera	*Amyelois transitella*	AtrFl(2)d	GenBank	XP_013189818	301	Complete
		Bombyx mori	BmFl(2)d	GenBank	XP_004931734	303	Complete
		Plutella xylostella	PxWTAP	GenBank	OQ291275	303	Complete
		Agrilus planipennis	AplFl(2)d	GenBank	XP_018327965	337	Complete
		Dendroctonus ponderosae	DpoFl(2)d	GenBank	XP_019758651	322	Complete
		Leptinotarsa decemlineata	LdFl(2)d	GenBank	XP_023019229	321	Complete
	Diptera	*Ceratitis capitata*	CcFl(2)d	GenBank	XP_020712578	652	Complete
		Lucilia cuprina	LcFl(2)d	GenBank	XP_023297132	585	Complete
		Musca domestica	MdFl(2)d	GenBank	XP_005177555	633	Complete
		Drosophila melanogaster	DmFl(2)d	GenBank	NP_001246305	412	Complete
	Hymenoptera	*Acromyrmex echinatior*	AeWTAP	GenBank	XP_011049880	465	Complete
		Apis mellifera	AmWTAP	GenBank	XP_006566329	467	Complete
		Bombus terrestri	BteWTAP	GenBank	XP_012169071	469	Complete
		Camponotus floridanus	CfWTAP	GenBank	XP_011258753	464	Complete
	Hemiptera	*Acyrthosiphon pisum*	ApiFl(2)d	GenBank	XP_008187985	391	Complete
		Diuraphis noxia	DnFl(2)d	GenBank	XP_015370775	339	Complete
		Cimex lectularius	CleFl(2)d	GenBank	XP_014244543	381	Complete

		Halyomorpha halys	HhFl(2)d	GenBank	XP_022173365	367	Complete
Spenito	Lepidoptera	*Bombyx mori*	BmSpenito	GenBank	XP_021206434	748	Complete
		Plutella xylostella	PxSpenito	GenBank	OQ291276	754	Complete
		Trichoplusia ni	TnSpenito	GenBank	XP_026743159	749	Complete
		Manduca sexta	MsSpenito	GenBank	XP_030031806	751	Complete
	Diptera	*Anopheles albimanus*	AaSpenito	GenBank	XP_035794344	798	Complete
		Hermetia illucens	HiSpenito	GenBank	XP_037906055	749	Complete
		Drosophila melanogaster	DmSpenito	GenBank	NP_001286174	793	Complete
	Hymenoptera	*Osmia bicornis*	ObSpenito	GenBank	XP_029037509	772	Complete
		Cephus cinctus	CciRBM15B	GenBank	XP_015597733	769	Complete
		Colletes gigas	CgSpenito	GenBank	XP_043257206	771	Complete
		Megachile rotundata	MrRBM15B	GenBank	XP_003704493	772	Complete
	Hemiptera	*Cimex lectularius*	ClRBM15	GenBank	XP_014243265	733	Complete
		Halyomorpha halys	HhRBM15B	GenBank	XP_014292768	737	Complete
		Nilaparvata lugens	NlSpenito	GenBank	XP_022190533	783	Complete
		Homalodisca vitripennis	HvSpenito	GenBank	XP_046661825	779	Complete
Virilizer	Lepidoptera	*Helicoverpa armigera*	HaVirlizer	GenBank	XP_021189381	1963	Complete
		Galleria mellonella	GmVirlizer	GenBank	XP_026762801	1824	Complete
		Plutella xylostella	PxVirlizer	GenBank	OQ291277	1895	Complete
		Danaus plexippus	DpVirlizer	GenBank	XP_032520943	1948	Complete
		Agrilus planipennis	ApVirlizer	GenBank	XP_018336095	1870	Complete
		Photinus pyralis	PpyVirlizer	GenBank	XP_031343309	1784	Complete
		Onthophagus taurus	OtVirlizer	GenBank	XP_022905685	1864	Complete
	Diptera	*Aedes albopictus*	AaVirlizer	GenBank	XP_029711671	1872	Complete
		Hermetia illucens	HiVirlizer	GenBank	XP_037919018	1804	Complete
		Drosophila melanogaster	DmVirlizer	GenBank	NP_524900	1854	Complete
	Hymenoptera	*Formica exsecta*	FeVirlizer	GenBank	XP_029676694	1486	Complete
		Camponotus floridanus	CfVirlizer	GenBank	XP_011253692	1489	Complete

		Harpegnathos saltator	HaslVirlizer	GenBank	XP_025154593	1486	Complete
	Hemiptera	*Homalodisca vitripennis*	HvVirlizer	GenBank	XP_046659594	1710	Complete
		Halyomorpha halys	HhVirlizer	GenBank	XP_024217806	1662	Complete
		Nilaparvata lugens	NlVirlizer	GenBank	XP_039284109	1968	Complete
Flacc	Lepidoptera	*Bicyclus anynana*	BaZC3H13	GenBank	XP_023941530	1005	Complete
		Bombyx mori	BmZC3H13	GenBank	XP_004930924	1013	Complete
		Plutella xylostella	PxFlacc	GenBank	OQ291278	960	Complete
	Coleoptera	*Anoplophora glabripennis*	AgZC3H13	GenBank	XP_018578427	1141	Complete
		Agrilus planipennis	ApFlacc	GenBank	XP_018324212	1179	Complete
		Photinus pyralis	PpyFlacc	GenBank	XP_031343424	1124	Complete
	Diptera	*Glossina fuscipes*	GfFlacc	GenBank	XP_037895652	1040	Complete
		Bactrocera oleae	BoFlacc	GenBank	XP_014085849	1093	Complete
		Drosophila melanogaster	DmFlacc	GenBank	NP_001285419	842	Complete
	Hymenoptera	*Leptopilina heterotoma*	LhZC3H13	GenBank	XP_043477161	1179	Complete
		Orussus abietinus	OaZC3H13	GenBank	XP_012279904	1306	Complete
		Cephus cinctus	CciZC3H13	GenBank	XP_015607734	1216	Complete
	Hemiptera	*Nilaparvata lugens*	NlZC3H13	GenBank	XP_039281391	1171	Complete
		Homalodisca vitripennis	HvZC3H13	GenBank	XP_046686487	1008	Complete
Hakai	Lepidoptera	*Bombyx mori*	BmHakai	GenBank	XP_004924335	361	Complete
		Plutella xylostella	PxHakai	GenBank	OQ291279	370	Complete
		Ostrinia furnacalis	OfHakai	GenBank	XP_028163025	366	Complete
		Manduca sexta	MsHakai	GenBank	XP_030035972	399	Complete
	Coleoptera	*Asbolus verrucosus*	AvHakai	GenBank	RZC36223	357	Complete
		Photinus pyralis	PpHakai	GenBank	XP_031333302	363	Complete
		Diabrotica virgifera	DvirgHakai	GenBank	XP_028146885	367	Complete
		Nicrophorus vespilloides	NvHakai	GenBank	XP_017770044	358	Complete
	Diptera	*Teleopsis dalmanni*	TdHakai	GenBank	XP_037958067	489	Complete
		Drosophila guanche	DgHakai	GenBank	XP_034134836	433	Complete

	Drosophila kikkawai	DkHakai	GenBank	XP_017033885	289	Complete
	Drosophila melanogaster	DmHakai	GenBank	NP_001260593	473	Complete
Hymenoptera	*Nylanderia fulva*	NfHakai	GenBank	XP_029154725	452	Complete
	Pseudomyrmex gracilis	PgHakai	GenBank	XP_020299762	458	Complete
	Camponotus floridanus	CfHakai	GenBank	EFN67678	415	Complete

Table S3. Primers used in CRISPR/Cas9 experiment

Purpose	Gene name	Primer name	Primer sequence (5´-3´)	PCR product (bp)	Tm (°C)
CRISPR sgRNA[a)]	m^{6}A-KO-sgRNA	CRISPR-SEKO-F1	*GAAATTAATACGACTCACTATAGG***TACCAGGACTGTATAGCTTG**GTTTTAGAGCTAGAAATAGC	124	70
		CRISPR-R	AAAAGCACCGACTCGGTGCCACTTTTTCAAGTTGATAACGGACTAGCCTTATTTTAACTTGCTATTTCTAGCTCTAAAAC		
CRISPR ssODN[b)]	ssODN	—	CTCTACAGCAACAGCTGAAGCGCCAGGGTACCAGG**C**CTGTATAGCTTGCTTGAGTTCTTACGGACCGATTGAAAACGGGTACAACACCCA	90	—
Identification of m^{6}A mutation[c)]	m^{6}A-KO	m^{6}A-KO-F	CGGGAGACTTCAGGTACCAG	204	55
		m^{6}A-KO-R	ACAATTTCCACACCACCGTG		

[a)]A specific oligonucleotide encoding a T7 polymerase-binding site (italicized) and an optimal sgRNA target sequence (underlined and bold) of the 3′-UTR region of *PxJHE* in *P. xylostella* resistance NIL-R strains were designed as the forward primer CRISPR-F, and a common oligonucleotide encoding the remaining sequences of CRISPR-F were designed as the reverse primer CRISPR-R.

[b)]The precise m^{6}A mutant site indicated with base C (bold), and conversion of PAM site "CGG" to "CTT" to prevent re-cleavage of Cas9 protein.

[c)]To identify the m^{6}A mutation at the 3′-UTR region of *PxJHE* in *P. xylostella* NIL-R strain, a specific primer pair (m^{6}A-KO-F/ m^{6}A-KO-R) was designed to amplify a 204-bp gDNA fragment surrounding sgRNA target site.

Table S4. CRISPR/Cas9-induced m[6]A mutation in *P. xylostella*

Original strain	G_0				G_1			G_2	Mutant strain
	Eggs[a)]	Hatched (%)	Adults (%)	Sib-cross[b)]	Pupae	Genotypes (%)[c)]	Sib-cross[d)]	Homozygotes (%)[e)]	
NIL-R	489	216/489 (44)	155/216 (72)	50♂× 50♀	144	40/144 (28)	—	—	m[6]A-KO
						55/144 (38)	—	—	
						25/144 (17)	11♂× 14♀	51	
						24/144 (17)	—	—	

a) A total of 489 fresh pre-blastoderm eggs from the *P. xylostella* NIL-R strain were collected for microinjection.

b) 50 male and 50 female moths from m[6]A-KO were randomly selected and sib-crossed to produce G_1 progeny.

c) A total of 144 pupae of G_0 were obtained for genotyping, four genotypes were included: wild-type homozygotes, 28% (40/144); HDR heterozygotes, 38% (55/144); HDR homozygotes, 17% (25/144); NHEJ heterozygotes, 17% (24/144).

d) 11 males and 14 female homozygotes were sib-mated to generate G_2 progeny.

e) 51 individuals of G_2 were homozygous for m[6]A mutation by sequencing.
